# The Repeated Replacement Method: A Pure Lagrangian Meshfree Method for Computational Fluid Dynamics

**DOI:** 10.1371/journal.pone.0039999

**Published:** 2012-07-06

**Authors:** Wade A. Walker

**Affiliations:** Austin, Texas, United States of America; German Cancer Research Center, Germany

## Abstract

In this paper we describe the repeated replacement method (RRM), a new meshfree method for computational fluid dynamics (CFD). RRM simulates fluid flow by modeling compressible fluids’ tendency to evolve towards a state of constant density, velocity, and pressure. To evolve a fluid flow simulation forward in time, RRM repeatedly “chops out” fluid from active areas and replaces it with new “flattened” fluid cells with the same mass, momentum, and energy. We call the new cells “flattened” because we give them constant density, velocity, and pressure, even though the chopped-out fluid may have had gradients in these primitive variables. RRM adaptively chooses the sizes and locations of the areas it chops out and replaces. It creates more and smaller new cells in areas of high gradient, and fewer and larger new cells in areas of lower gradient. This naturally leads to an adaptive level of accuracy, where more computational effort is spent on active areas of the fluid, and less effort is spent on inactive areas. We show that for common test problems, RRM produces results similar to other high-resolution CFD methods, while using a very different mathematical framework. RRM does not use Riemann solvers, flux or slope limiters, a mesh, or a stencil, and it operates in a purely Lagrangian mode. RRM also does not evaluate numerical derivatives, does not integrate equations of motion, and does not solve systems of equations.

## Introduction

In this paper, we first present background material on CFD and discuss previous CFD methods which have informed this work. Then we motivate RRM and explain its workings in depth. Next, we show that RRM gives correct results for many standard test problems. We also demonstrate that RRM shows steadily decreasing error in its solutions as we increase the desired accuracy, and that RRM handles many common types of boundary conditions. Finally, we discuss the similarities and differences between RRM and other CFD methods.

### Background

CFD is the use of numerical methods to model liquid and gas flow. CFD has many practical uses, from the analysis of the airflow over vehicles to the design of water turbines.

CFD covers a vast range of fluid compositions and flow types. For simplicity, we only consider a fluid that is:

- **Continuous**: Infinitely subdividable, unlike real fluids which are made of discrete atoms and molecules.
- **Simple**: Completely described by density, velocity, and pressure at each point, which we call the “primitive variables”, and write as *ρ*, *u*, and *p*. We do not consider other possible fluid properties like chemical reactivity. We also do not consider the action of non-pressure forces like gravity or electromagnetism on the fluid.
- **Ideal**: Described by the ideal gas law, in which the internal energy of a fluid is purely a function of *ρ*, *p*, and *γ*. The constant *γ* is called the ratio of specific heats, and has a value of about 1.4 for air.
- **Single-phase**: Consisting entirely of either liquid or gas, but not a mixture of the two. This means we need not model liquid-gas interfaces. We also do not consider the interaction of solid objects with the fluid.
- **Inviscid**: Having no resistance to deformation. This simplifies the equations of fluid motion.
- **Adiabatic across contacts**: Allowing no heat to flow from one side of a contact discontinuity to the other. This means that contact-adjacent regions will not tend towards the same temperature. We compare RRM’s results to fluid flows that are adiabatic across contacts because of the availability of analytic solutions, but we show later that RRM is not adiabatic across contacts.
- **One-dimensional**: Having only one spatial dimension. This makes illustration and programming simpler.

**Figure 1:**
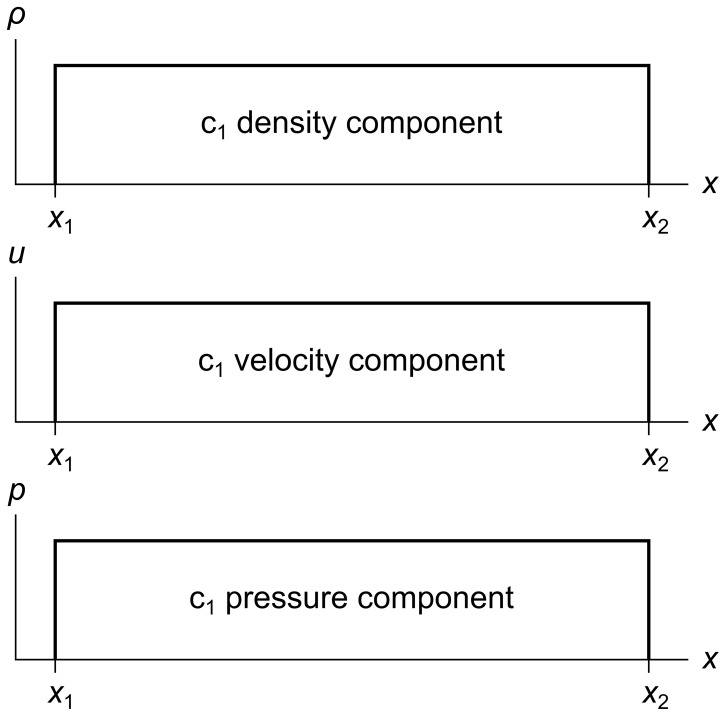
Fluid cell with three separate components. Fluid cell c_1_ has density, velocity, and pressure components *ρ*, *u*, and *p*. The left and right coordinates of the cell are *x*_1_ and *x*_2_.

Even though our fluid is infinitely subdividable, for illustration and analysis we divide it into finite-sized cells. Figure 1 shows a cell c_1_ with its left edge at *x*_1_ and its right edge at *x*_2_. The density, velocity, and pressure components are shown on separate graphs.

**Figure 2:**
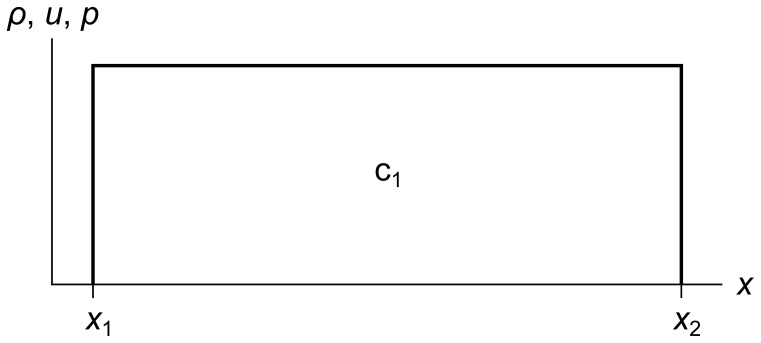
Fluid cell with three superimposed components. Fluid cell c_1_ has density, velocity, and pressure components all superimposed on the same axis. The left and right coordinates of the cell are *x*_1_ and *x*_2_.

When we do not need to show all three components separately, we combine them onto one axis for simplicity as shown in figure 2, with the understanding that *ρ*, *u*, and *p* may have different values even though they are drawn with the same line.

We can describe fluid flow with cells in two main ways. The Eulerian description considers the cells to be stationary, and the fluid to flow across their edges and through them. The Lagrangian description considers the cells to move along with the fluid, so any given bit of fluid is always found in the same cell. We will initially use the Eulerian description since it is the most common. We will later switch to the Lagrangian description when we describe RRM in more detail.

Given the restrictions and cell definition above, we can model fluid flow with a set of equations called the Euler equations, which can be derived from the local conservation of mass, momentum, and energy. The Euler equations take on different forms depending on whether we write them for the Eulerian or Lagrangian description of fluid flow. For the Eulerian description, we write the Euler equations in English like this:

- **Conservation of mass**: The mass in a cell changes by the amount that flows across its edges.
- **Conservation of momentum**: The momentum in a cell changes by the amount that flows across its edges, and by the amount due to the pressure acting on its edges.
- **Conservation of energy**: The energy in a cell changes by the amount that flows across its edges, and by the amount due to work done by the pressure acting on its edges.

The Euler equations are typically written as partial differential equations, but we write them here as integral equations because it is more natural for our derivative-free numerical method. Here are the Euler equations for a cell, written for the Eulerian description of fluid flow, in conservation form:
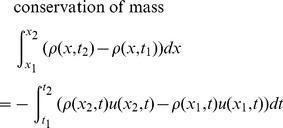


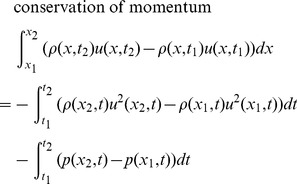
(1)

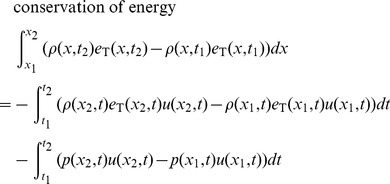



The coordinates *x*_1_ and *x*_2_ are the left and right edges of the cell. The times *t*_1_ and *t*_2_ are the starting and ending times of a period where fluid is flowing into and out of the cell, and pressure is acting on the cell edges.

This form is called the conservation form because it is written in terms of the conserved quantities per unit length. These conserved quantities are mass per unit length *ρ*, momentum per unit length *ρu*, and energy per unit length *ρe*_T_.

The specific total energy *e*_T_ is the energy per unit mass due to both macroscopic and microscopic motion. The ideal gas law gives us equations for *e*_T_ and for the speed of sound *a*, which we will use later.
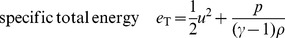
(2)

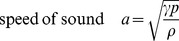
(3)


To write the Euler equations in a more compact form we define a vector of the conserved quantities
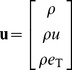
(4)and a vector of the fluxes (plus the pressure and pressure work) at the edges



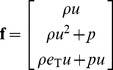
(5)Then the Euler equations can be written as a single vector equation
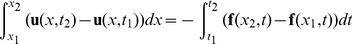
(6)


**Figure 3:**
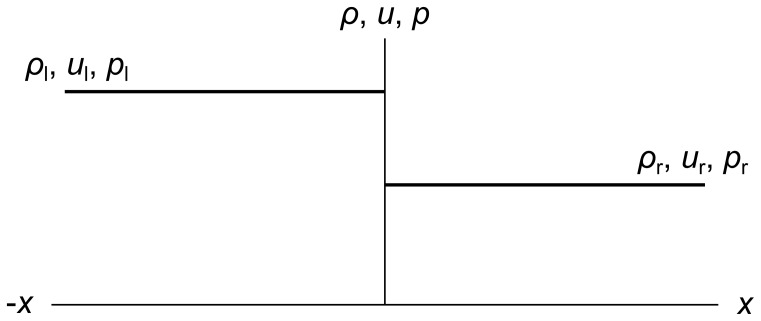
The Riemann problem. The Riemann problem specifies initial density, velocity, and pressure values of *ρ*_l_, *u*_l_, *p*_l_ on the left side of the origin and *ρ*_r_, *u*_r_, *p*_r_ on the right side of the origin.

For the general initial conditions *ρ*(*x*,*t*_1_), *u*(*x*,*t*_1_), and *p*(*x*,*t*_1_), the Euler equations have no known analytical solution. This is inconvenient when we wish to check the results of a numerical method. So in this paper we restrict ourselves to simple initial conditions known as the Riemann problem, where *ρ*, *u*, and *p* take on the constant values (*ρ*_l_, *u*_l_, *p*_l_) and (*ρ*_r_, *u*_r_, *p*_r_) on the left and right sides of an initial discontinuity, as shown in figure 3.

Unlike the general initial conditions, the Riemann problem has an analytical solution, though this solution contains a nonlinear implicit equation and a number of special cases that we must treat carefully. In this paper, we use a Riemann solver due to Toro [1] as a standard to test RRM’s results against. Many CFD methods, beginning with Godunov’s method in 1959 [2], use an embedded Riemann solver as a part of their algorithms, though RRM does not.

Even for the Riemann problem, accurate numerical solutions to the Euler equations are challenging, mainly because the solutions can include discontinuities. At these discontinuities, the spatial derivatives in the differential form of the Euler equations are undefined, which spoils many simple numerical methods and requires special-case code in more advanced methods.

In the solutions to many other partial differential equations such as the heat equation, initial discontinuities will smear out and become increasingly smooth over time. But in the solutions to the Euler equations, initial discontinuities do not always smear out, and indeed new discontinuities may arise over time.

**Figure 4:**
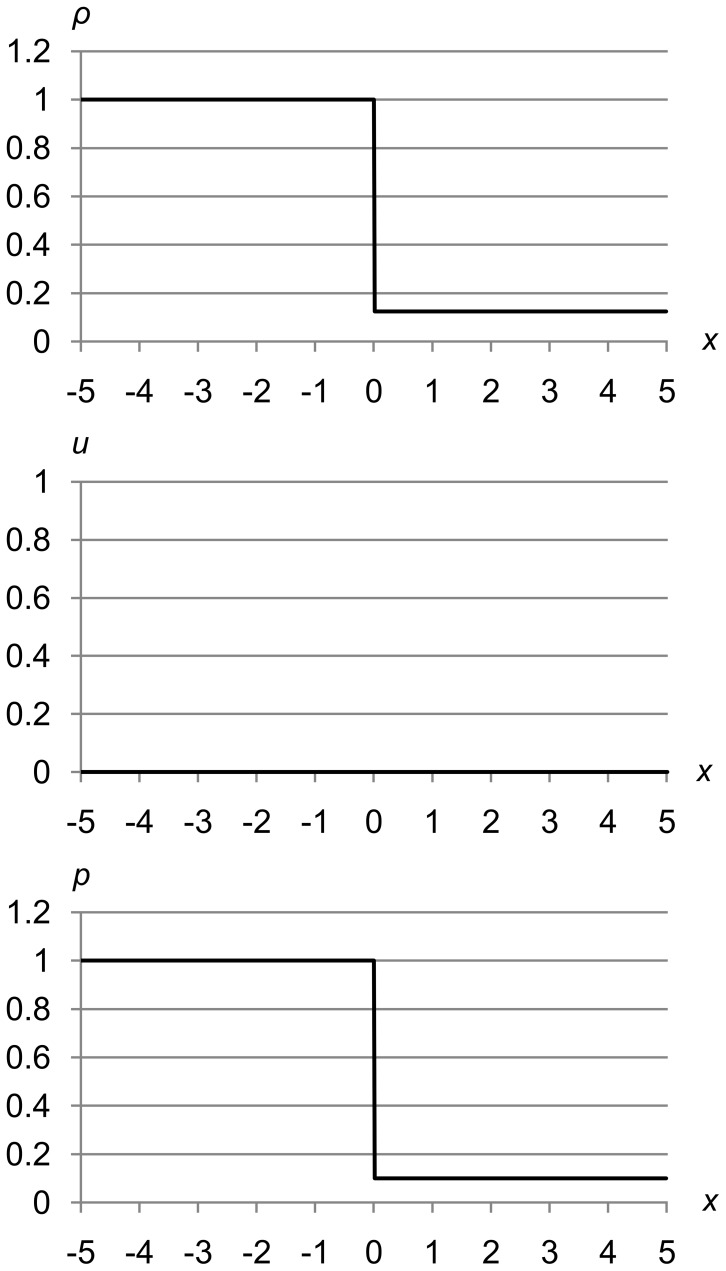
Sod’s shock tube problem at *t* = 0.0 seconds. Sod’s shock tube problem showing initial density, velocity, and pressure values (*ρ*_l_, *u*_l_, *p*_l_) = (1.0, 0.0, 1.0) and (*ρ*_r_, *u*_r_, *p*_r_) = (0.125, 0.0, 0.1).

For example, consider Sod’s shock tube problem [3], a special case of the Riemann problem. A shock tube is a gas-filled tube with a diaphragm in the center. The diaphragm is initially airtight, so the left and right sides of the tube can be separately charged to specific pressures and densities as shown in figure 4, which sets (*ρ*_l_, *u*_l_, *p*_l_) = (1.0, 0.0, 1.0) on the left side, and (*ρ*_r_, *u*_r_, *p*_r_) = (0.125, 0.0, 0.1) on the right side.

**Figure 5:**
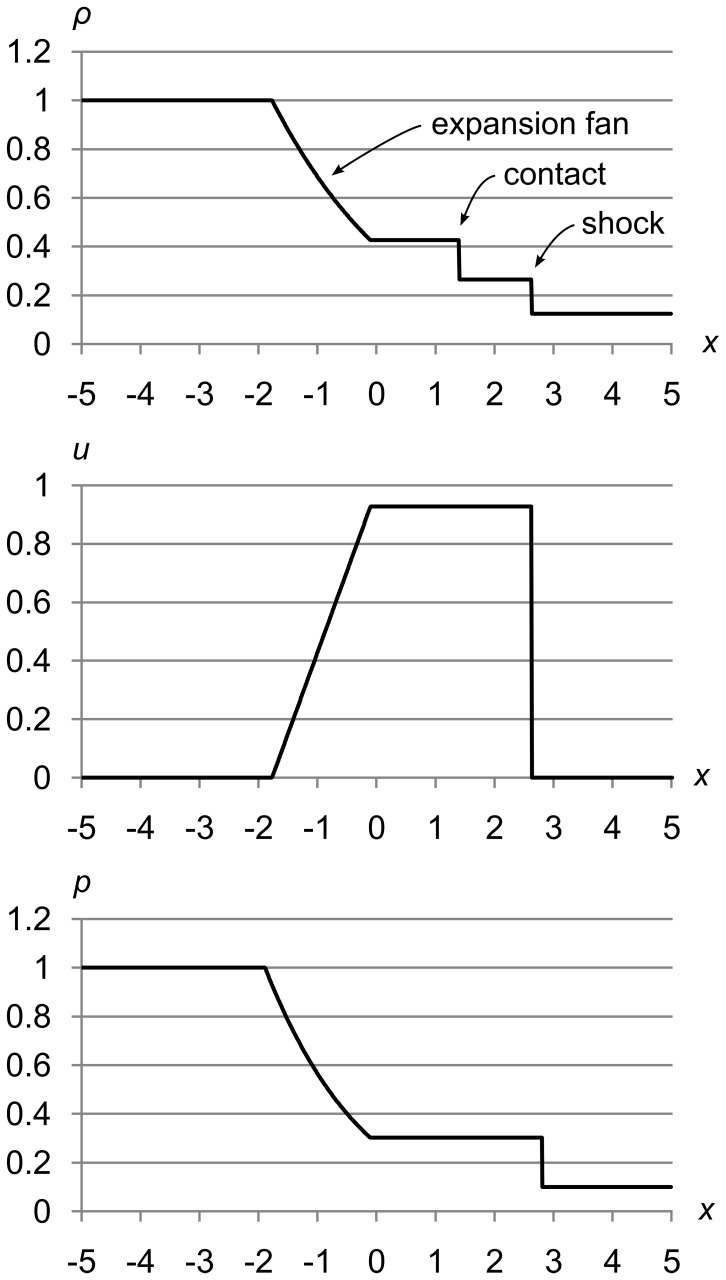
Sod’s shock tube problem at *t* = 1.5 seconds. Sod’s shock tube problem showing density *ρ*, velocity *u*, and pressure *p* after 1.5 seconds of time evolution. We can see three flow features: an expansion fan, a contact, and a shock.

At time *t* = 0.0, we instantly remove the diaphragm and let the fluid start flowing from left to right. Figure 5 shows the fluid at *t* = 1.5 seconds. We can see both types of discontinuity that are possible in solutions to the Euler equations, as well as the “expansion fan” that joins the high-pressure left state to the flat area in the center.

The first type of discontinuity, a contact, separates two areas that differ only in density. Contacts travel along with the fluid, and since velocity is constant across a contact, no fluid flows across them. Contacts cannot form spontaneously; they must either be present in the initial conditions as in Sod’s problem, or they must be formed by the intersection of two shocks. As a real-world example, if stationary hot and cold water masses are carefully placed side by side, they will be separated by a contact discontinuity, at least until heat energy flows across the discontinuity and smears it out.

The second kind of discontinuity, a shock, can be formed by a pressure gradient steep enough to force the fluid to move faster than the local speed of sound *a*. Shocks can develop over time, and need not be present in the initial conditions. Density, velocity, and pressure can all change across a shock. As a real-world example, if you pilot a boat through the water faster than waves can travel through the water, the boat creates a shock at its bow.

**Figure 6:**
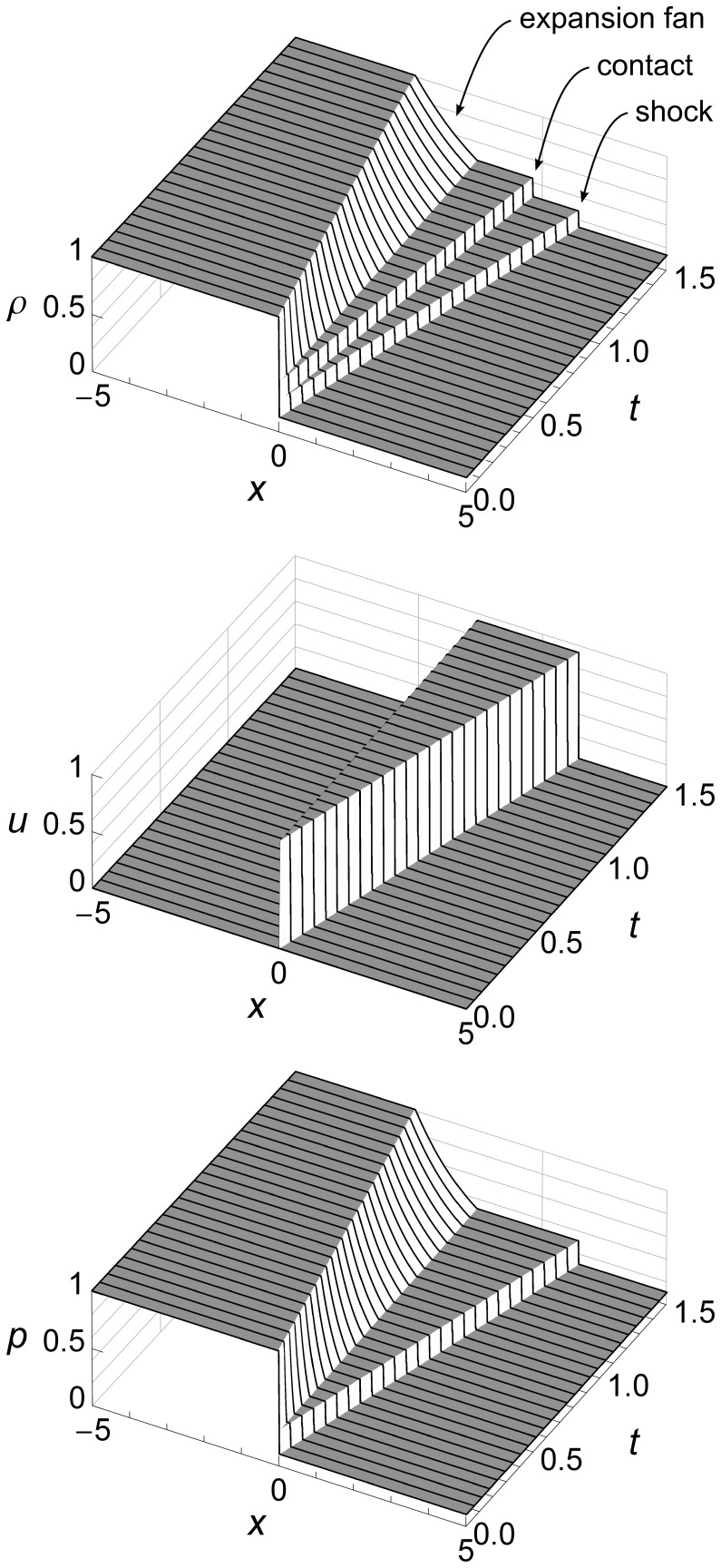
Sod’s shock tube problem time evolution from *t* = 0 to *t* = 1.5 seconds. Sod’s shock tube problem showing density *ρ*, velocity *u*, and pressure *p* from time *t* = 0 to time *t* = 1.5 seconds. We can see three flow features: an expansion fan, a contact, and a shock. The contact and the shock both start at the origin and move to the right, with the shock running ahead due to its higher speed. The expansion fan gradually slopes left as more and more fluid flows to the right to feed the shock.

The 2D graphs of Sod’s shock tube problem above show flow features at specific times, but do not show how the fluid flow evolves over time. Figure 6 uses 3D to add a time dimension.

These graphs show how the contact and the shock both start at the origin and move to the right, with the shock running ahead due to its higher speed. They also show how the expansion fan gradually slopes left as more and more fluid flows to the right to feed the travelling shock.

For subsequent figures we will mainly use 2D graphs, since they allow easier comparison of our results with those of a Riemann solver. We will use 3D only when the time evolution of the flow is of special interest, such as when we illustrate boundary conditions.

### Previous Work

If you simply use the definition of the derivative to convert the Euler equations from differential equations to algebraic equations, you get the finite difference method (FDM). In conservation form, FDM models a fluid as a set of cells, each of which contains the values of the conserved quantities at a point within the cell. The explicit version of FDM calculates those values at the next time step from the values in nearby cells at the current time step. The set of nearby cells is called the stencil.

The finite volume method (FVM) also models a fluid as a set of cells, but it stores cell average values instead of point samples in the cells. In its explicit conservation form, FVM calculates the values at the next time step by adding and subtracting fluxes of the conserved quantities across each neighboring cell’s edges during the time step.

The finite element method (FEM) was historically used for structural mechanics [4], but began to find use in fluid dynamics [5] as the method was generalized and applied to time-varying problems. FEM starts by creating a mesh of elements (cells in our terminology) which are shaped to fit the problem geometry. FEM then solves a system of equations at each time step to determine the unknown fluid values in each element. Fluid values in FEM are typically stored in piecewise polynomial form, as opposed to the point samples of FDM or the cell averages of FVM.

In FDM and FVM, the fluid is usually considered to move through stationary cells in a single, global coordinate system. This is the Eulerian description of fluid flow mentioned above.

**Figure 7:**
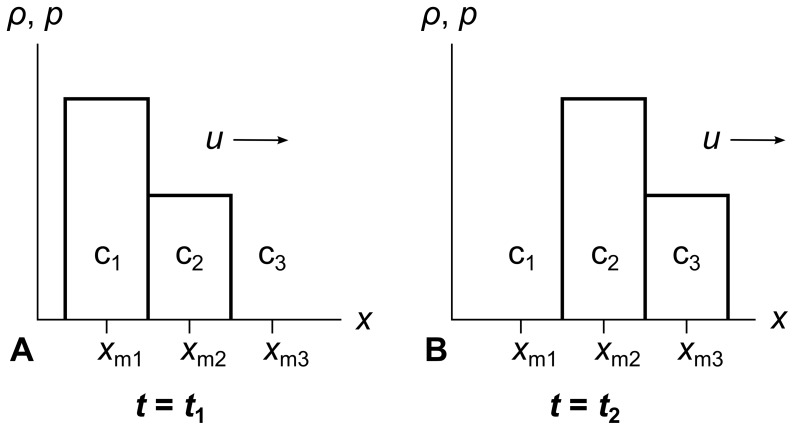
Eulerian description of fluid flow. In the Eulerian description of fluid flow, the fluid moves through stationary cells. Consider a global coordinate system divided into three cells c_1_, c_2_, and c_3_. The fluid is traveling rightwards with velocity *u*. In panel A at time *t*_1_, a measurement at point *x*_m1_ will show the density *ρ* and pressure *p* of cell c_1_. In panel B at a later time *t*_2_ = (*x*_m2_– *x*_m1_)/u, we measure the same density and pressure at point *x*_m2_ because the fluid has moved to the right by one cell width.

Figure 7 shows an example with three stationary cells c_1_, c_2_, and c_3_. The measuring points *x*_m1_, *x*_m2_, and *x*_m3_ are at the cell centers. The entire fluid has a rightward velocity *u*. In panel A at time *t*_1_, we measure cell c_1_’s *ρ* and *p* values at *x*_m1_, and c_2_’s values at *x*_m2_. Cell c_3_ is empty.

In panel B at a later time *t*_2_ = (*x*_m2_– *x*_m1_)/*u*, all the fluid from c_1_ has moved into c_2_, and all the fluid from c_2_ has moved into c_3_. Now we measure the same *ρ* and *p* values at *x*_m2_ that we previously measured at *x*_m1_, and the same values at *x*_m3_ that we previously measured at *x*_m2_. The fluid has moved one cell width to the right, but the cells themselves have stayed in place.

Eulerian methods are relatively simple to implement, but shocks, contacts and other steep gradients may smear out or gain unphysical oscillations as they cross cell edges, depending on the algorithm used. Researchers have proposed many refinements over the years to increase accuracy, such as Total Variation Diminishing (TVD) methods [6], Essentially Non-Oscillatory (ENO) methods [7], Monotone Upwind Schemes for Scalar Conservation Laws (MUSCL) [8], the Piecewise-Parabolic Method (PPM) [9], and many more.

Another approach to Eulerian fluid flow is the lattice Boltzmann method (LBM) [10]. Instead of a mesh of cells, LBM uses a lattice of connected sites, each of which can “stream” fluid to a fixed number of neighboring sites. Each site contains a distribution function that represents how much fluid is streaming in each direction. After each streaming step, LBM executes a “collision” step at each site to alter the distribution functions to maintain conservation. LBM has many attractive features, including ease of programming and simple handling of boundary conditions.

In contrast to FDM, FVM, and LBM, FEM often uses the alternative Lagrangian description of fluid flow, in which the cells travel along with the fluid.

**Figure 8:**
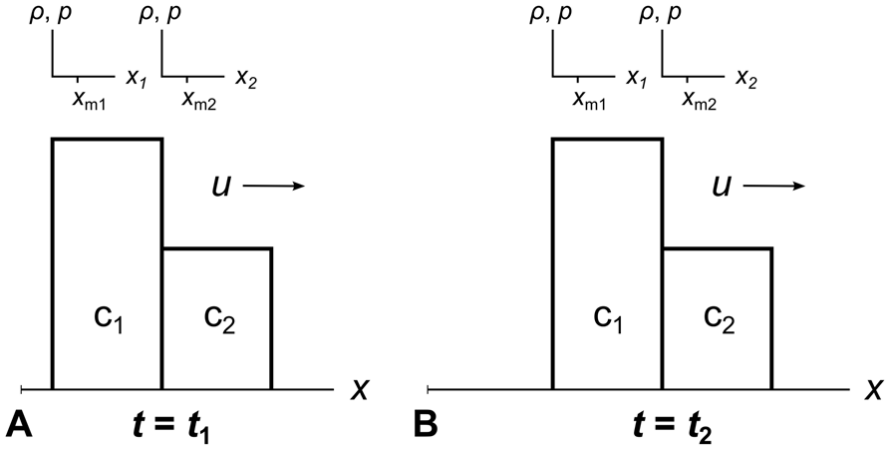
Lagrangian description of fluid flow. In the Lagrangian description of fluid flow, the cells are part of the fluid and move along with it. Consider fluid cells c_1_ and c_2_ traveling with their own local coordinate systems. The cells and their coordinate systems are both traveling rightwards at velocity *u* in the global coordinate system. In panel A at time *t*_1_, and in panel B at any later time *t*_2_, points *x*_m1_ and *x*_m2_ in the cells’ coordinate systems remain at the same places in those cells. No fluid crosses cell edges.

Figure 8 shows an example, with two cells c_1_ and c_2_ moving to the right with a velocity *u*, similar to the Eulerian example above. However, in the Lagrangian description the fluid does not move across cell edges. Instead, the cells themselves move, carrying local coordinate systems along with them.

Panel A shows us measuring the values at time *t*_1_ of *ρ* and *p* at point *x*_m1_ in the local coordinate system of cell c_1_. Panel B shows that we will measure the same values at any later time *t*_2_, since cell c_1_ and its coordinate system move together. The same holds true for cell c_2_ in its local coordinate system at its own point *x*_m2_.

Lagrangian methods handle shocks and contacts naturally, because those flow features travel with the fluid instead of smearing out as they cross cell edges. But pure Lagrangian methods are rare, because as the fluid flows, the cells can become excessively bunched up, stretched out, or deformed, which can reduce simulation accuracy and efficiency.

The cells of FDM, FVM, and FEM, and the lattice sites of LBM, are usually connected in a mesh. Each cell has a well-defined shape, and each cell or site has a fixed set of neighbors. In simple methods, these shapes and sets of neighbors are constant over the whole course of the simulation. But in Eulerian methods, a fluid may have complex flow features that move around over time, so we may want to create smaller cells in those complex areas and larger cells in other areas. Or in Lagrangian methods, some cells may become degenerate or singular in a complex flow, so that the method’s equation solver no longer works correctly.

**Figure 9:**
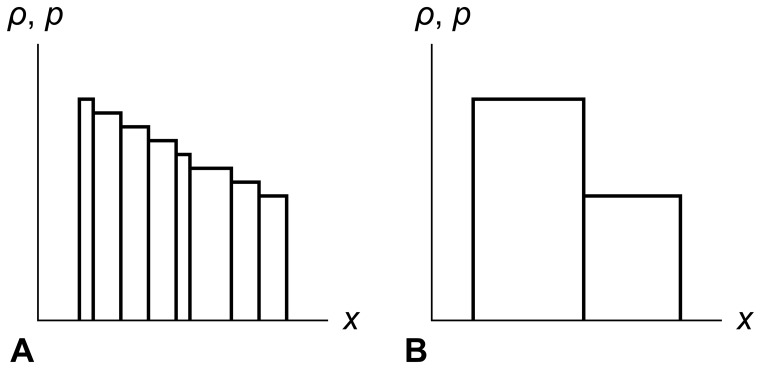
Remeshing. The eight cells in panel A can be remeshed into two cells in panel B that cover the same area and contain the same mass, momentum, and energy. Some CFD methods require remeshing to maintain accuracy or to prevent numerical difficulties.

The process of changing the mesh to alleviate these problems is called remeshing. Figure 9 panel A shows eight small fluid cells, and panel B shows those eight cells remeshed into two cells that cover the same area.

To avoid this complication, the so-called meshfree methods do away with mesh connectivity entirely. One of the first meshfree methods was smoothed-particle hydrodynamics (SPH) [11], [12]. SPH is a purely Lagrangian method which models a fluid with a set of moving particles, and computes the fluid’s properties at any point by summing the contributions of nearby particles using a kernel function which smooths out the particles’ properties over some “smoothing length”. SPH was originally motivated by the study of astrophysical problems such as galaxy formation, where the constituents were already discrete particles. SPH was later applied to other problems where the fluid was presumed to be continuous before being discretized.

The moving-particle semi-implicit method (MPS) [13] is a meshfree method similar to SPH, which was originally intended for simulation of incompressible fluids with interacting free surfaces. It also uses a kernel function (called a weight function in the MPS literature), but one which is specially designed to repel particles at short distance, thereby maintaining approximately constant density in the fluid. MPS has been applied to many situations, including simulations of coastal waves and dam breaks.

Much research in meshfree methods has been done in recent years, and there is now a great variety of such methods with different kernel functions, particle properties, and integration techniques. Li and Liu [14] and Huerta et al. [15] have both written excellent surveys of the field.

## Methods

### Motivation

**Figure 10:**
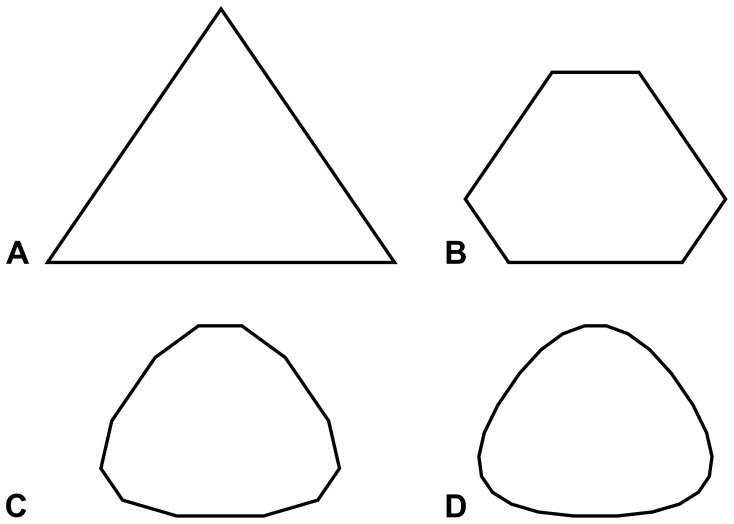
Chaikin’s corner-cutting algorithm. Starting with the triangle in panel A, cutting the corners off one-quarter of the way along each side gives us panel B. Panels C and D show the process carried out two more times. We can repeat this process until the curve has any desired smoothness.

RRM was motivated by Chaikin’s corner-cutting algorithm for curve generation, which evolved into the subdivision curves of computational geometry [16], [17]. A curve of this type starts as a set of lines joined end to end to form a roughly faceted curve, shown in figure 10 panel A. First we cut off each of the corners one-quarter of the way along each side, shown in panel B. Then we cut the corners off the new corners, shown in panel C, iteratively refining the curve into smaller and smaller line segments, until a desired level of smoothness is reached in panel D.

RRM does the same sort of iterative refinement, but on a moving fluid instead of a stationary curve, and with constraints on conservation of mass, momentum and energy rather than constraints on surface continuity and smoothness.

### Overview

**Figure 11:**
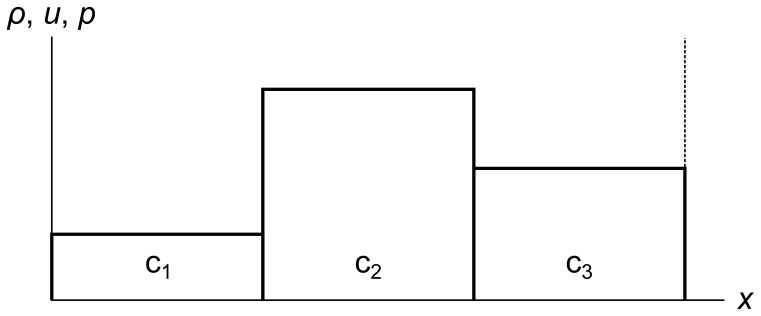
Fluid cells. A fluid divided into three cells c_1_, c_2_, and c_3_. The dotted line at the right shows that there are periodic boundary conditions, so the right side of c_3_ is adjacent to the left side of c_1_.

To begin, we divide a fluid into finite-sized cells. In one dimension, each cell is a line segment with an associated density, velocity, and pressure, all of which are constant across the cell. Figure 11 shows a fluid divided into three cells c_1_, c_2_, and c_3_. For now we use periodic boundary conditions, so the left side of c_1_ is adjacent to the right side of c_3_. We indicate this with the dotted line on the right of c_3_.

**Figure 12:**
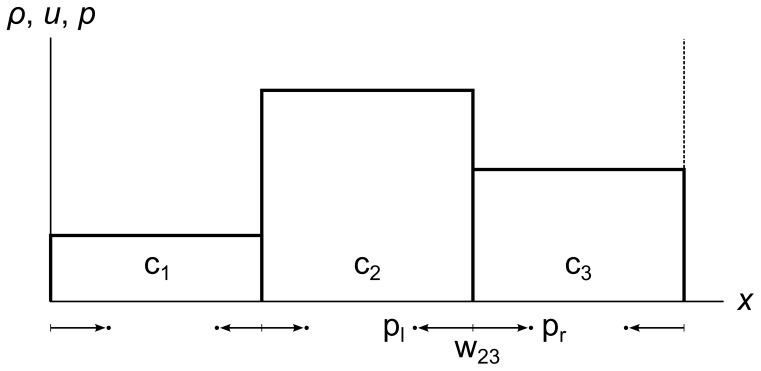
Fluid cells showing wavefronts and tracer particles. A fluid divided into three cells c_1_, c_2_, and c_3_. The dotted line at the right shows that there are periodic boundary conditions. Wavefront w_23_ originates between c_2_ and c_3_, and contains tracer particles p_l_ and p_r_ that travel through the fluid at the local speed of sound *a* = sqrt(*γp*/*ρ*). The constant *γ* depends on the fluid (it has a value of 1.4 for air). The *ρ* and *p* values are those of the cell the particle is traveling through. Note that w_31_ (not labeled) extends into both c_3_ and c_1_ due to the periodic boundary conditions.

At each cell edge, we send tracer particles left and right through the fluid at the local speed of sound *a*, as shown in figure 12. Each pair of tracer particles defines an expanding wavefront of change that originates at the cell edge. For example, in figure 12 we show w_23_, the wavefront originating between c_2_ and c_3_, along with its left tracer particle p_l_ and its right tracer particle p_r_.

**Figure 13:**
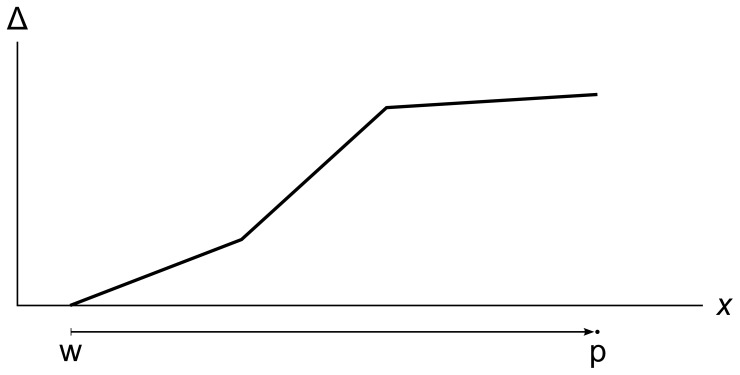
Error metric growing as a tracer particle travels. A particle p traveling right as part of wavefront w. Its error metric Δ increases as the particle travels, with the slope of Δ changing at cell edges.

As each tracer particle travels, it accumulates an error metric that tracks how much each of the primitive variable values has changed, and over what distance. Figure 13 shows a tracer particle p, the right-hand particle of wavefront w, moving through the fluid. The particle’s error metric Δ grows as the particle moves, with the slope of Δ changing when the particle crosses each cell edge.

When this error metric for either of the two tracer particles in any wavefront exceeds a preset maximum, we chop the wavefront area out of the fluid, flatten the chopped-out cell parts into a single new cell, and insert that new cell into the hole left by the chopping.

In areas of the fluid where primitive variable values differ greatly from cell to cell, tracer particles’ error metrics will accumulate quickly, so new cells will be chopped out soon after wavefront creation. This leads to more, smaller cells in areas of the fluid with steep slopes. Conversely, in areas where values are very similar from one cell to the next, error metrics will accumulate slowly, so we will chop out fewer, larger cells in areas of the fluid with shallow slopes.

**Figure 14:**
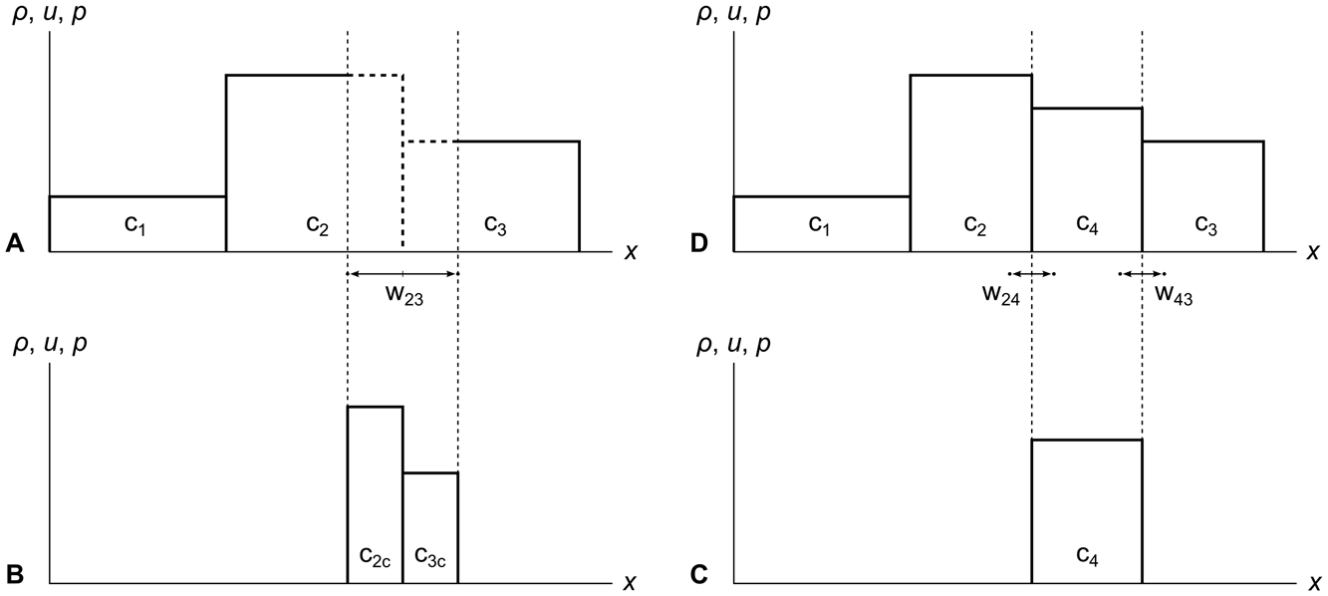
Chopping, flattening, and new cell creation. Panel A shows the chopping of wavefront w_23_ out of the fluid, which chops off the right side of c_2_ and the left side of c_3_. Panel B shows the resulting chopped parts c_2c_ and c_3c_. Panel C shows the flattening of the two chopped parts into a new cell c_4_ with the same mass, momentum, and energy. Panel D shows the insertion of the new cell c_4_ into the fluid and the creation of the new wavefronts w_24_ and w_43_ on its edges.

We illustrate this whole process in figure 14. In panel A, we chop wavefront w_23_ out of the fluid, removing the wavefront’s tracer particles from the fluid at the same time. This leaves us with chopped cell parts c_2c_ and c_3c_, shown in panel B. Panel C shows us flattening c_2c_ and c_3c_ into a new cell c_4_ of the same mass, momentum and energy. Then in panel D we insert c_4_ into the fluid and create new wavefronts w_24_ and w_43_ at the cell edges.

The chop-flatten-create process always results in exactly one new cell, and always shrinks two other cells by chopping parts off of them. But this process can also remove any number of whole cells if the maximum error metric allows the wavefront to grow wide enough. For example, if wavefront w_23_ in figure 14 had grown wider, it could have chopped off the right side of c_1_, entirely removed c_2_, and chopped off the left side of c_3_, resulting in no net change in the number of cells. An even wider wavefront which removes two whole cells would reduce the total number of cells in the fluid by one, and so on. This is how RRM increases and decreases the total number of cells over time to adapt to changing fluid conditions.

The last step in the RRM algorithm is to choose the next wavefront whose tracer particles have reached the maximum error metric and repeat the chop-flatten-create process detailed above. This repetition evolves the fluid simulation forward in time.

### Stored Quantities

In each cell, we store three main types of data:

- The size, shape, and position of the cell. In one dimension, cells have only width, so we need only store the time-varying *x* coordinate *x*_1_(*t*) of the cell’s left edge, and the cell’s width *w*.
- The cell’s three primitive variable values *ρ*, *u*, and *p*.
- Four extra vector quantities which help us ensure conservation.

Below we explain the relationships between these quantities and show how to derive other necessary values from them.

RRM is purely Lagrangian and represents the fluid as finite-sized cells, so we use the integral Lagrangian form of the Euler equations, written in terms of the primitive variables:
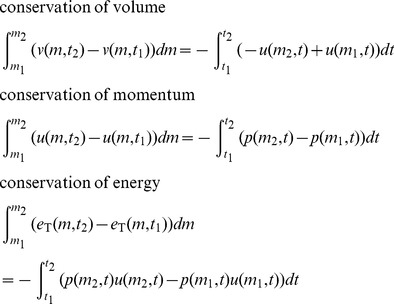
(7)


The values *m*_1_ and *m*_2_ are the mass coordinates of the left and right side of the cell. The mass coordinates move with the fluid, unlike the fixed spatial coordinates *x*_1_ and *x*_2_ that we used in the Eulerian form of these equations in equation set 1. This means that the fluid between *m*_1_ and *m*_2_ stays between *m*_1_ and *m*_2_, with no fluid flow across the cell edges. We can get the mass coordinate *m* at a point in the fluid from the Eulerian coordinate *x* at that point by integrating all the mass up to that point:
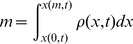
(8)


So the value of the mass coordinate at any point is the sum of all the mass to the left of that point in the fluid.

Note that the conservation of mass equation does not appear in equation set 7. That is because the mass between *m*_1_ and *m*_2_ is constant, so that equation would be trivial. Instead we show the conservation of volume equation, which merely says that a cell’s volume *v* changes as its edges move towards or away from each other. In the Eulerian form of these equations, it was the conservation of volume equation that was trivially equal to a constant, so we omitted it from equation set 1.

Note also that the equations for the conservation of momentum and energy are simpler in the Lagrangian form than in the Eulerian form. This is because we do not need the flux terms that describe momentum and energy flowing across the cell edges, now that the cell edges move with the fluid.

As we saw with the Eulerian form, if we define a vector of the conserved quantities
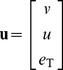
(9)and a vector of the velocity, pressure and pressure work at the cell edges
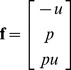
(10)then the Lagrangian form of the Euler equations can be written as a single vector equation



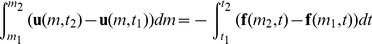
(11)We do not store the cells’ conserved quantities directly, but we can calculate them by integrating over the primitive variables. Since our primitive variables are piecewise constant, the integrals are simply multiplications by *w*, the width of the cell.
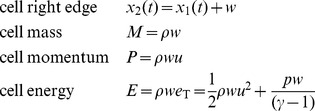
(12)


To allow our flattening process to exchange energy between kinetic and potential forms while remaining conservative, we store two extra vector quantities on each edge of each cell: pressure momentum and pressure energy.

**Figure 15:**
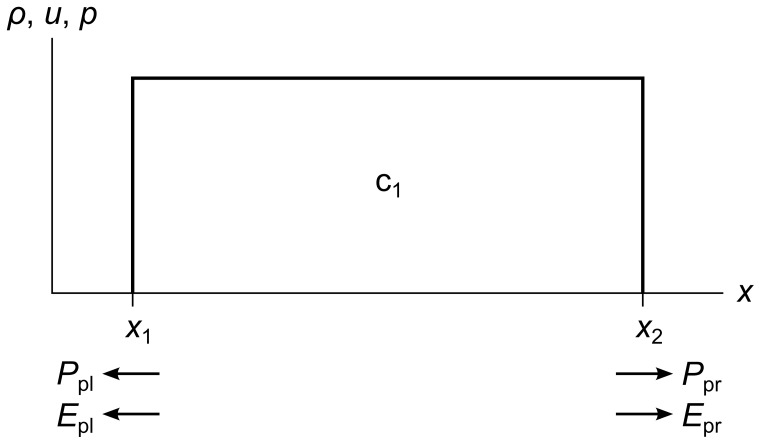
Pressure momentum and pressure energy vectors. A single cell c_1_ showing left and right pressure momentum *P*_pl_ and *P*_pr_, and left and right pressure energy *E*_pl_ and *E*_pr_. Portions of these vectors are transferred to new cells during the chopping and flattening process, and this transfer is what causes the conversion between potential and kinetic energy and vice versa.



Figure 15 shows these quantities for a single cell c_1_. We define them as follows:

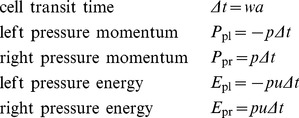
(13)The first quantity Δ*t* is the time it takes a tracer particle to cross a cell, and also the time it takes for a cell to completely expend its store of pressure momentum and pressure momentum upon its neighbors. The next four quantities are the terms on the right-hand sides of the momentum and energy Euler equations from equation set 7. They represent the changes in momentum and energy due to pressure work that a cell has the potential to cause to its neighbors. We store equal quantities of *P*_p_ and *E*_p_ in each direction, so for each cell they sum to zero, leaving the overall momentum and energy of the fluid unchanged.

As the fluid evolves, the total fluid mass, momentum, and energy remain strictly constant when these extra *P*_p_ and *E*_p_ vectors are summed along with the cells’ mass, momentum, and energy.
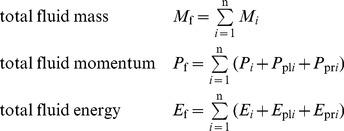
(14)


This insures strict conservation of mass, momentum, and energy over the course of the simulation.

### Cell Chopping and Flattening

**Figure 16:**
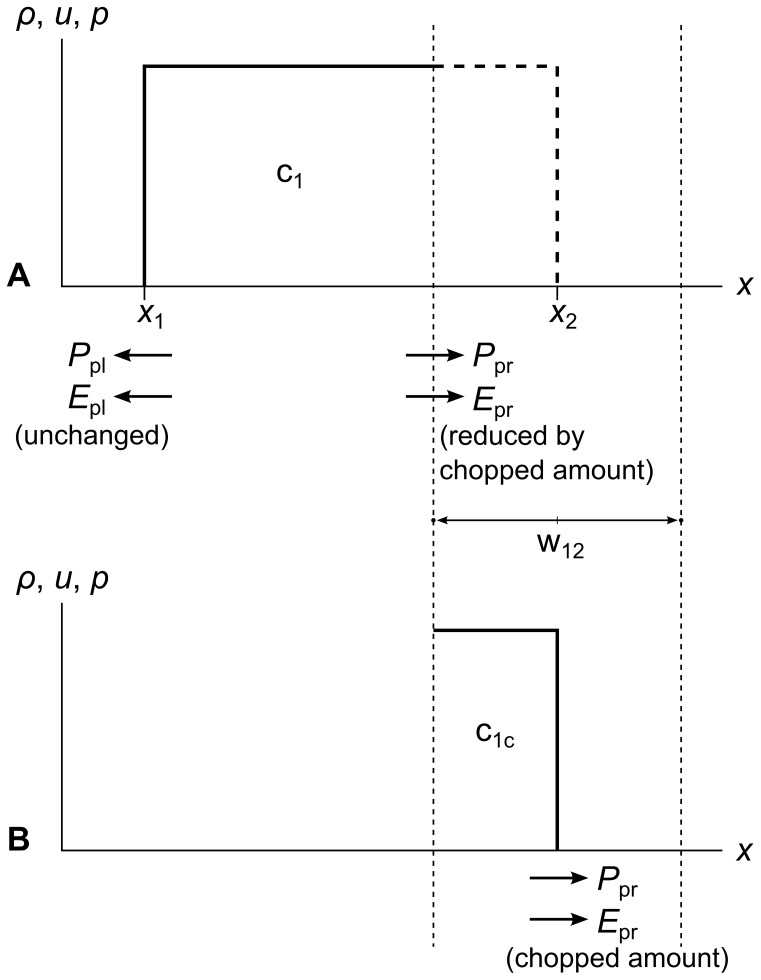
Chopping pressure momentum and pressure energy vectors. Panel A shows a single cell c_1_ with the right side chopped off. Panel B shows the chopped mass, momentum, energy, pressure momentum *P*_p_, and pressure energy *E*_p_ that are now contained in the chopped cell part c_1c_, which will be flattened into a new cell along with any other cells chopped out by the same wavefront. The amounts of mass, momentum, and energy transferred to c_1c_ are proportional to the width of c_1c_, but the amounts of pressure momentum and pressure energy transferred to c_1c_ are proportional to the time since the creation of c_1_.

When we chop off one side of a cell, we are removing five quantities: mass, momentum and energy, plus part of the pressure momentum and pressure energy from the vectors on the chopped-off edge of the cell. Figure 16 shows this for a single cell c_1_. Panel A shows the quantities remaining in c_1_ after chopping, and panel B shows the quantities that are chopped off to form cell part c_1c_.

Note that we chop off mass, momentum and energy in amounts proportional to the width of the chopped part, but we chop off pressure momentum and pressure energy in an amount proportional to the time since the chopped cell was created. This is because mass, momentum and energy are inherent properties of the fluid that must be integrated over space, whereas pressure momentum and pressure energy act over time to convert energy from potential to kinetic form when there is a gradient in the fluid pressure. We can see this in equation set 7, where the left-side integrals are spatial, and the right-side integrals are temporal. In RRM, we treat *P*_p_ and *E*_p_ as acting steadily over time, starting at time *t*_c_ when a cell is created, and ending at time *t*_c_+Δ*t*, the time at which both of the cell’s tracer particles (the left wavefront’s right particle, and the right wavefront’s left particle) leave the cell.

When chopping out a new cell, we first determine its intersections with existing cells. Then we chop off those intersections and add up all the mass *M*, momentum *P*, and energy *E* the intersections contained. Then, using the width of the new cell *w* and the requirement that density, velocity, and pressure are constant across it, we can calculate the flattened values of the primitive variables for the new cell.
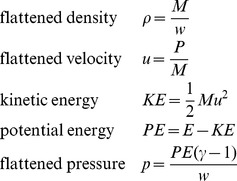
(15)


### Negative Pressure Fix

Very occasionally, the flattening process will produce a cell with negative pressure, either because of rounding or truncation error, or because a very small wavefront chops a large amount of pressure momentum and pressure energy, which would accelerate the newly created small cell more than its store of potential energy can support.

When this happens, we flatten the cell without adding in the chopped pressure momentum and pressure energy, which turns off pressure-to-momentum conversion for that cell and gives us a positive pressure after flattening. The unused pressure momentum and pressure energy are added to that of the newly created cell, which spreads the pressure-to-momentum conversion out over a slightly longer time.

Whitehurst’s signal method [18] uses a similar fix for negative pressures, but averages over space instead of time. When negative pressure occurs in a cell, the signal method averages that cell’s mass, momentum and energy with its three neighbors, in proportion to their volumes.

### Tracer Particles and Their Error Metric

The movement of the tracer particles through the cells of the fluid models the movement of characteristics or acoustic wavefronts through the fluid. The tracer particles do not represent real physical particles, they are merely a computational device. They do not carry mass, momentum, or energy, they do not interact with each other, and they do not affect cells’ properties. They always travel at the local speed of sound *a* in the cell that contains them.

As the tracer particles move through the fluid, we accumulate an error metric that tells us when to stop and chop out a new cell. The error metric **Δ**_1,n_ is the error accumulated by a tracer particle as it travels from cell 1 to cell n.
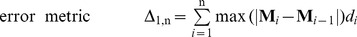
(16)where *d_i_* is the distance the tracer particle travels in cell *i*, and the metric vector **M** *_i_* for cell *i* is
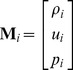
(17)When **Δ**_1,n_ for either tracer particle exceeds a user-supplied Δ^max^, we chop out a new cell.

This error metric needs a bit of explaining. First, the metric is a vector of all the primitive variables (instead of choosing just one or two) so that variation in any of them across the fluid can trigger the chopping of a new cell. So our maximum error metric **Δ**_max_ is a vector **Δ**_max_ = [Δ_max *ρ*_, Δ_max *u*_, Δ_max *p*_]^T^, with each value set separately by the user. When we say that **Δ**_1,n_ exceeds **Δ**_max_, we mean that some element of **Δ**_1,n_ exceeds the corresponding element of **Δ**_max_.

We take the absolute value of **M** *_i_* – **M** *_i_*_–1_ so the error metric will increase monotonically as the tracer particle travels. If we did not, the error metric might go up and down many times without exceeding **Δ**_max_, which could lead to chopping out new cells that contain more total variation in the primitive variable values than we meant to allow.

We multiply the error metric by distance so that the error metric grows even as the tracer particles move across cells, not just as the particles cross cell edges. This prevents us from chopping out unduly large new cells in areas of shallow density, velocity, or pressure gradients.

There is also a special case in this formula. When *i* is 1, **M**_0_ is the metric vector of the cell on the other side of the edge from where the tracer particle was created. So the tracer particle does not travel through cell 0, but its metric vector contributes to the overall error metric.

**Figure 17:**
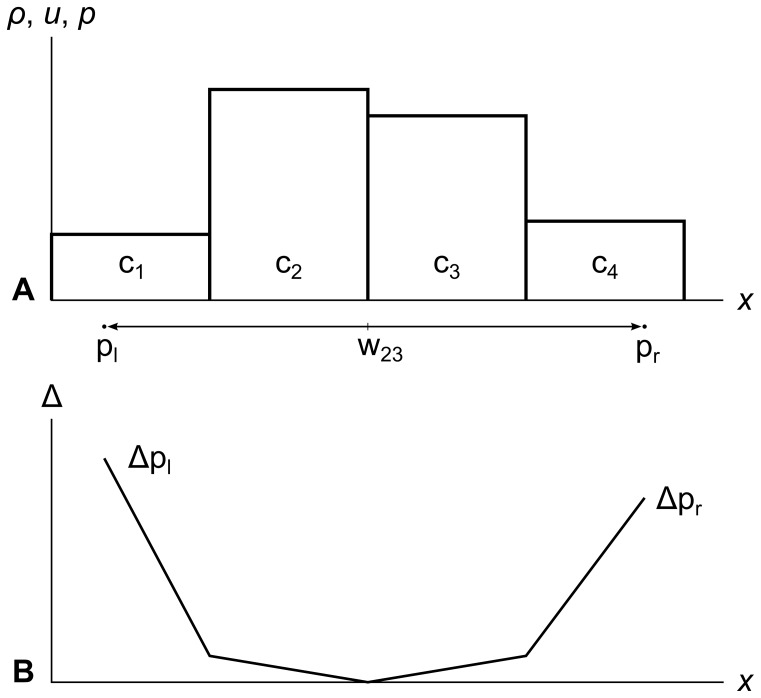
Error metric growing as particles cross cell edges. Panel A shows two particles p_l_ and p_r_ traveling through the fluid as part of wavefront w_23_. Panel B shows the particles’ error metrics Δp_l_ and Δp_r_ growing as the particles travel, and demonstrates how the error metric of each particle in a wavefront is tracked separately. Note how the slope of the error metric across each cell is proportional to the difference in the cells’ density *ρ*, velocity *u*, and pressure *p* at the edge the particle crossed to get into the cell.

Figure 17 panel A shows two tracer particles p_l_ and p_r_ traveling through a fluid as part of wavefront w_23_. Panel B shows how the error metrics Δp_l_ and Δp_r_ of the two particles change as the particles travel.

Note that the slopes of Δp_l_ and Δp_r_ are shallow in the center of the graph, because the density, velocity, and pressure of c_2_ and c_3_ are similar. As the particles cross into c_1_ and c_4_, the slopes of Δp_l_ and Δp_r_ increase substantially, which means that w_23_ will reach **Δ**_max_ sooner than it would have with a shallower gradient in the fluid.

### Wavefront Unioning

**Figure 18:**
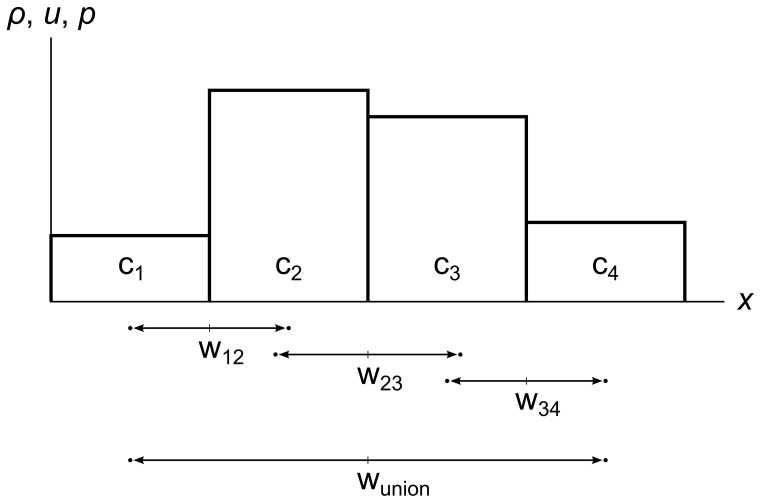
Wavefront unioning. A fluid divided into four cells c_1_, c_2_, c_3,_ and c_4_. Wavefront w_12_ overlaps wavefront w_23_, which overlaps wavefront w_34_, so we must chop out the union wavefront w_union_ to properly account for the effects of each wavefront on the others.

When we choose a wavefront that we wish to chop the fluid with, we first must check for overlap with other wavefronts. The final area we chop out will be the union of the first wavefront with all the wavefronts that overlap it, and all the wavefronts that overlap them, and so on. Figure 18 shows an example: if we choose w_12_, we see that it overlaps w_23_, which overlaps w_34_, so the final area we would have to chop is w_union_.

Wavefront unioning was motivated by the observation that once two expanding wavefronts overlap, the fluid in each one has affected the fluid in the other, so they can no longer be treated separately.

Wavefront unioning turns out to be essential for the stability of the simulation. Without wavefront unioning, it is possible to chop out an area that contains unbalanced pressure momentum and pressure energy, even in a perfectly “flat” fluid that has no density or pressure gradient. This imbalance can cause a newly-created cell’s velocity to be abnormally high, which causes a glitch in the simulation where fluid cells pile up or spread out in an unphysical way.

**Figure 19:**
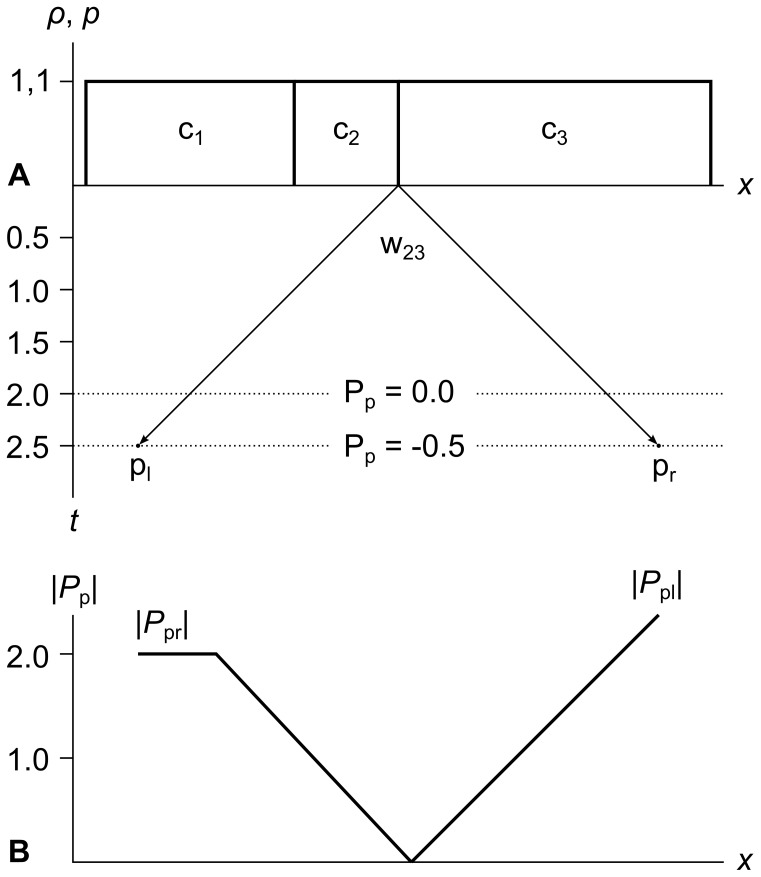
The “213 problem”. Panel A shows a fluid divided into three cells c_1_, c_2_, c_3_ of widths 2, 1, and 3 from left to right (hence the name “213 problem”). All three cells were created at time *t* = 0, and all three have density *ρ* = 1, pressure *p* = 1, and velocity *u* = 0. In a simulation without wavefront unioning, if wavefront w_23_ chopped out a new cell at time *t* = 2.5, that new cell would have a net momentum of −0.5. Panel B shows that this is because the rightward momentum *P*_pr_ from c_1_ levels off at *t* = 2.0, while the leftward momentum from c_3_ continues to increase until *t* = 3.0. This demonstrates that wavefront unioning is required to avoid unphysical changes in cell velocity during simulation.

Consider figure 19 panel A, which shows three cells c_1_, c_2_, and c_3_ with *ρ* = 1, *u* = 0, and *p* = 1. We call this the “213 problem” because the widths of the cells are 2, 1, and 3 from left to right.

Assume that all three cells were created at time *t* = 0, and that the speed of sound *a* = 1. Since there is no density or pressure gradient, chopping out a new cell anywhere in this fluid should result in a new cell with *ρ* = 1, *u* = 0, and *p* = 1.

Let us consider wavefront w_23_ expanding from the right side of c_2_ and see if this is true. At time *t* = 2, w_23_ will contain equal and opposite amounts of pressure momentum from c_1_ and c_3_, since *P*_pr1_ = 2 and *P*_pl3_ = −2. The pressure momenta *P*_pl2_ and *P*_pr2_ from c_2_ will cancel since the whole cell is covered, so the overall pressure momentum *P*_p_ = *P*_pr1_+*P*_pl2_+*P*_pr2_+*P*_pl3_ contained in w_23_ is zero, as shown by the dotted line at *t* = 2. So far, so good.

At time *t* = 2.5, the pressure momentum *P*_pr1_ from c_1_ is still 2, since it ran out of pressure momentum to contribute at *t* = 2. But the pressure momentum *P*_pl3_ from c_3_ is −2.5, since it will not run out until time *t* = 3.0. Figure 19 panel B shows how the wavefront’s left and right pressure momenta *P*_pl_ = *P*_pl2_+*P*_pl3_ and *P*_pr_ = *P*_pr1_+*P*_pr2_ increase as the wavefront expands, with *P*_pr_ leveling off at *t* = 2 when *P*_pr1_ stops increasing.

So if we chop out a new cell at time *t* = 2.5, it will have an overall pressure momentum *P*_p_ of −0.5, as shown by the dotted line at *t* = 2.5. This will make the new cell move to the left, even though there is no pressure gradient in the fluid! Unioning w_23_ with w_12_ (not shown) fixes this problem.

The analysis of the 213 problem shows that if we run a simulation without wavefront unioning, it will show occasional unphysical glitches. Since mass, momentum, and energy are all strictly conserved, the glitches sometimes smooth out over time, but if a glitch is big enough, it may create a large local gradient and significantly slow down simulation. Wavefront unioning avoids this problem.

### Discrete Event Simulation

RRM uses a discrete event simulation flow. We keep a priority queue of events, sorted in order of increasing event time. There are two kinds of events: particle events, where particles intersect cells, and wavefront events, where one of the particles in the wavefront exceeds the maximum error metric.

Particle events merely transfer particles from their current cell to the intersected cell, which changes their speed and the rate at which they accumulate error. Wavefront events chop new cells out of the fluid.

**Figure 20:**
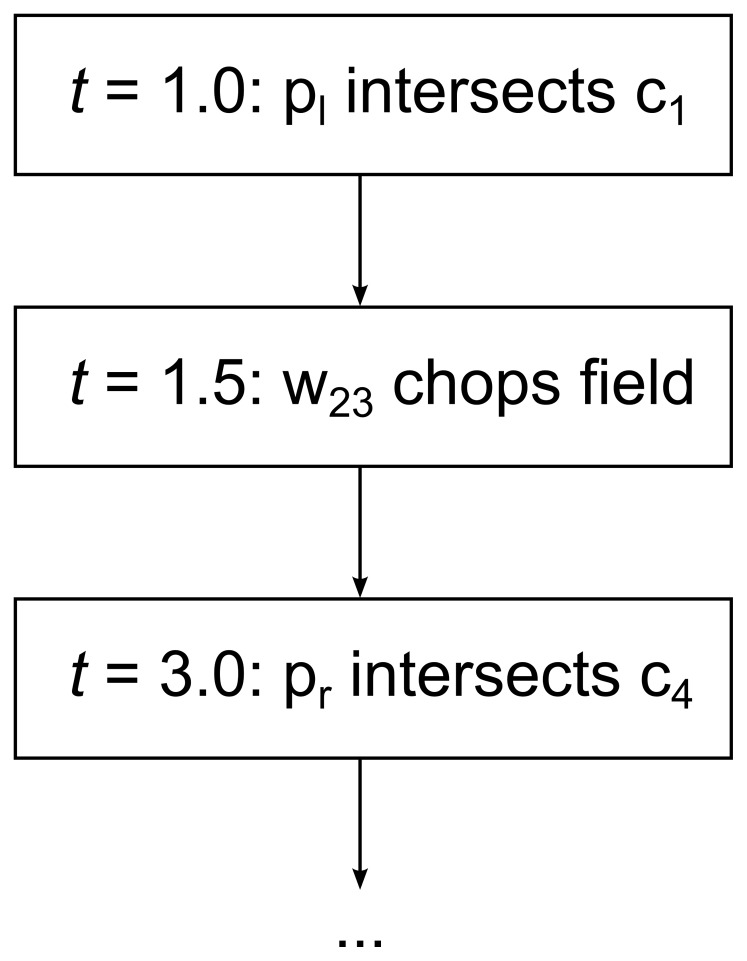
Simulation event queue. Queue of events associated with wavefront w_23_ in the previous example of the 213 problem. Events are ordered by increasing time, and the simulator always executes the event at the head of the queue.

Figure 20 shows what the event queue might look like for the previous example of the 213 problem at time *t* = 0, if we assume that the wavefront w_23_ would chop out a new cell at time *t* = 1.5. For simplicity, we show only the events associated with wavefront w_23_. In a real simulation there would be a wavefront between each pair of cells, so the event queue would be much more cluttered.

The first event transfers particle p_l_ from c_2_ to c_1_. The second event uses wavefront w_23_ to chop a new cell out of the fluid. The third event is removed at the same time the second event is processed, since a wavefront’s tracer particles are removed in the chopping process.

**Figure 21:**
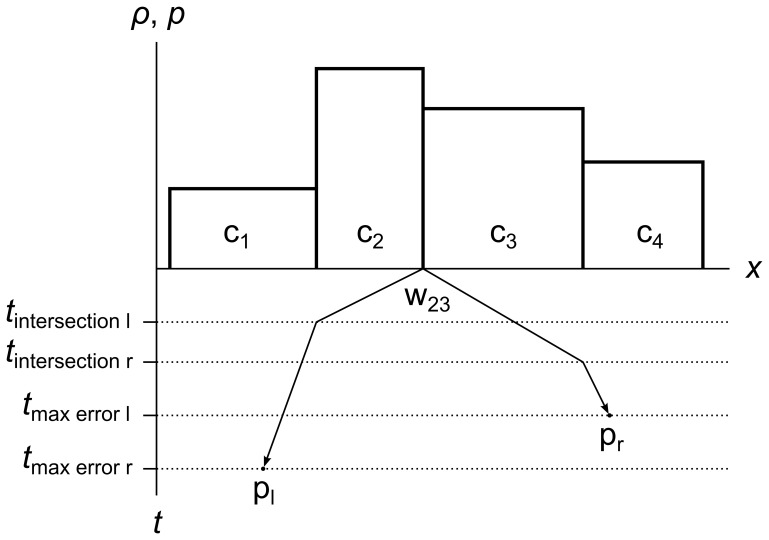
Particle intersection time and maximum error time. This figure shows all four possible event times for a single wavefront. Consider a fluid divided into four cells c_1_, c_2_, c_3,_ and c_4_. Wavefront w_23_ contains particles p_l_ and p_r_. Particle p_l_ intersects cell c_1_ at time *t*_intersection l_, and reaches the maximum error metric at *t*_max error l_. Particle p_r_ intersects cell c_4_ at time *t*_intersection r_, and reaches the maximum error metric at *t*_max error r_.

Here is how we determine the event times. When we create a wavefront with its pair of tracer particles, we find the intersection time *t*_intersection_ of each particle with the nearest cell edge in its direction of travel, and the time *t*_max error_ when the error metric of each particle will exceed the maximum error metric. Figure 21 shows all four of these times for particles p_l_ and p_r_ in wavefront w_23_.

We enqueue the two tracer particles as events, using the intersection times as the event times. We also enqueue the wavefront as an event, using the soonest of the max error times as the event time.
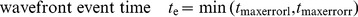
(18)


Each time we pull an event off the event queue, we check whether the event is a particle intersecting a cell, or a wavefront whose particle is reaching the maximum error metric. If a particle has intersected a cell, we transfer it into the intersected cell, recalculate the event time, and requeue the particle.

If either particle in a wavefront has reached the maximum error metric, we union the wavefront with any overlapping wavefronts, chop and flatten the area of the union into a new cell, and insert the new cell into the fluid. Then we create a new wavefront for each edge of the new cell and insert the corresponding events into the event queue. Finally, we transfer any particles caught in the chopped-out area into the new cell, which changes their speeds to the local speed of sound in the new cell, recalculates their event times, and requeues them.

### RRM Algorithm Flowchart

**Figure 22:**
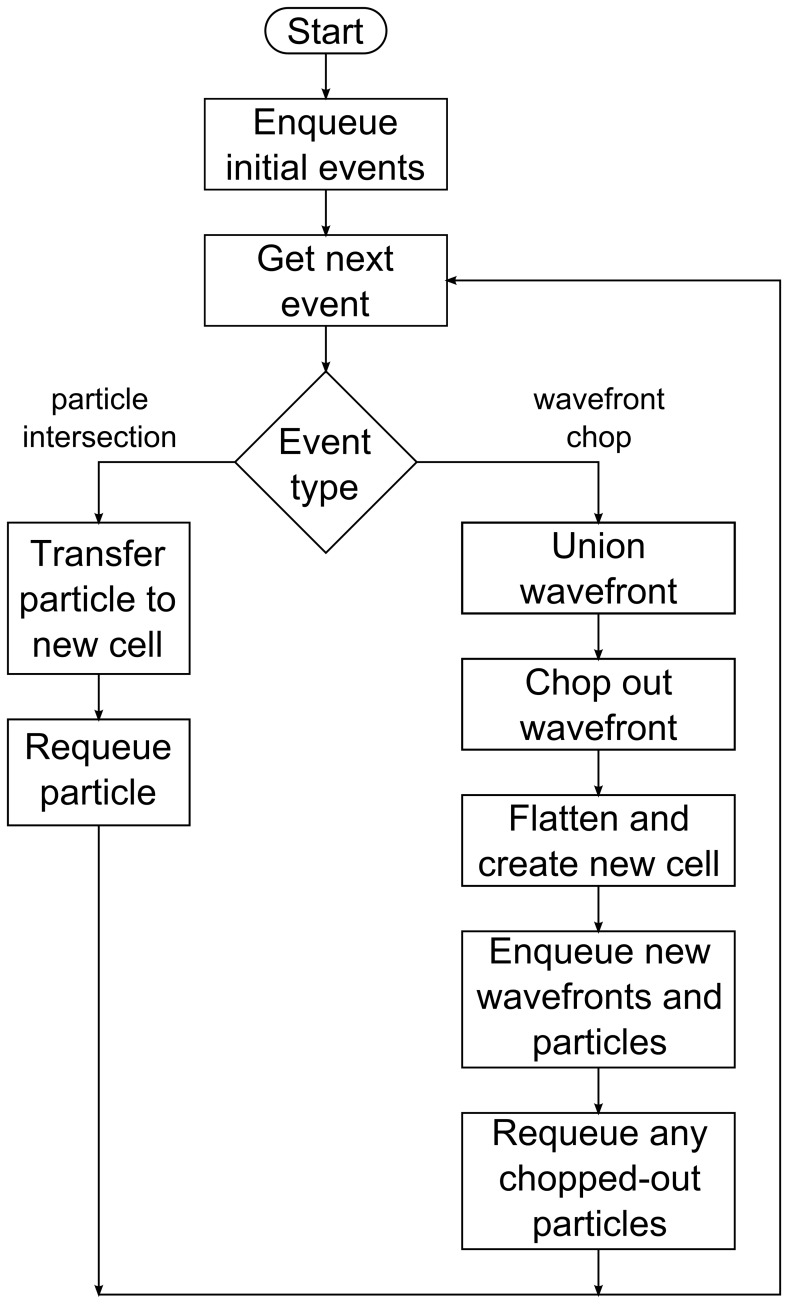
RRM flowchart. Flowchart showing the outline of the complete RRM algorithm.

For reference, figure 22 is a flowchart that outlines the entire RRM algorithm.

## Results

We tested RRM on nine standard test problems, and plotted RRM’s results (solid lines) against the output of Toro’s Riemann solver (dashed lines). The two match closely in most cases, with some exceptions discussed below.

In the following tests, RRM typically uses a maximum of 200 to 400 cells during the simulation, depending on the maximum error metric we set. Most of those cells are concentrated in areas of high gradient, with only a few wide cells in flat areas. We set the maximum error metrics to obtain good results in a relatively short time. In the error analysis section that follows these test results, we will justify our choices of these maximum error metrics and show how the quality of the results varies as the maximum error metrics are varied.

### Test 1

**Figure 23:**
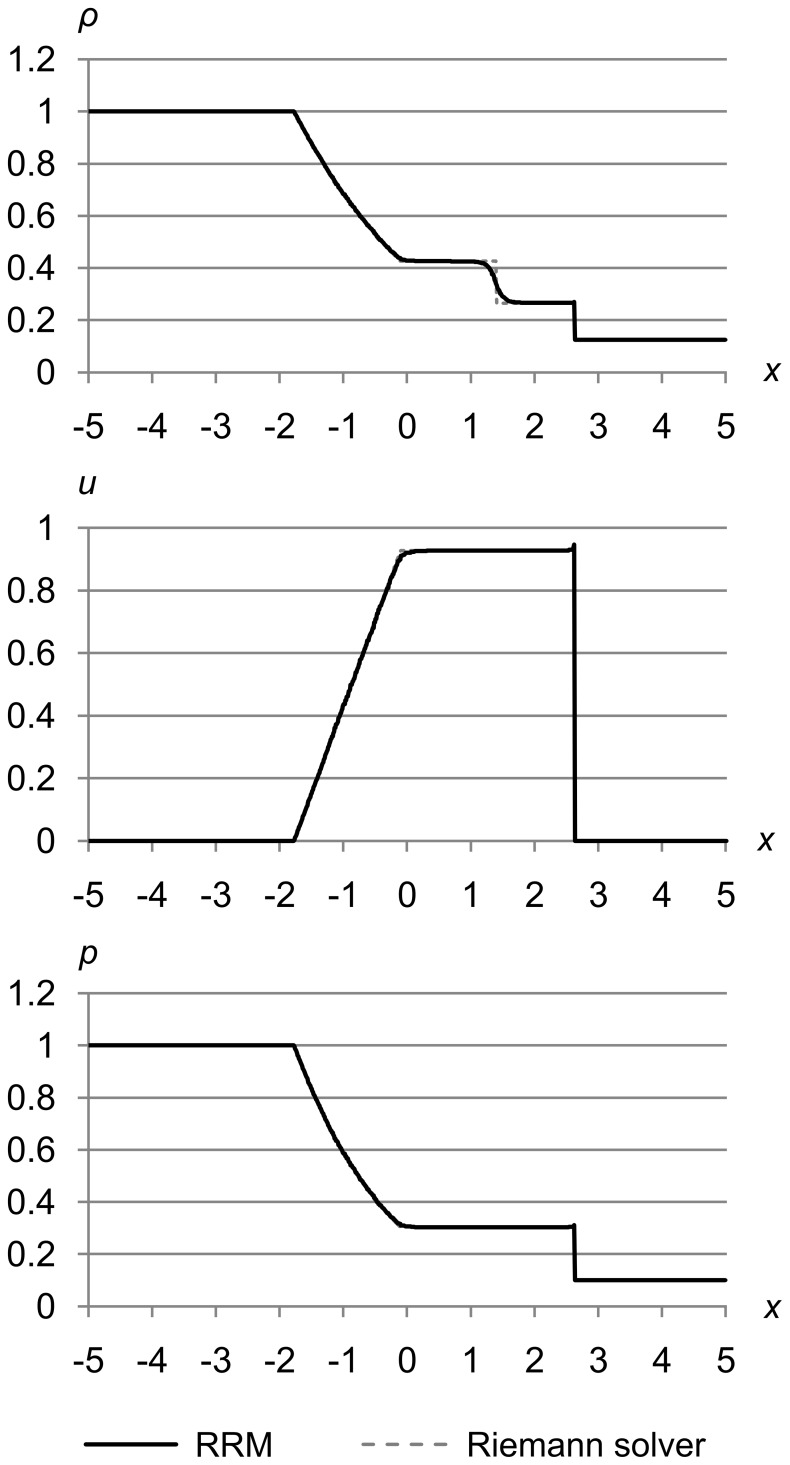
Test 1: Sod’s test problem at high accuracy. Sod’s problem with initial conditions (*ρ*_l_, *u*_l_, *p*_l_) = (1.0, 0.0, 1.0) and (*ρ*_r_, *u*_r_, *p*_r_) = (0.125, 0.0, 0.1), with maximum error metric (Δ_max *ρ*_, Δ_max *u*_, Δ_max *p*_) = (1.0e-5, 1.0e-3, 1.0e-3), at time *t* = 1.5. This test shows typical RRM results: an s-shaped contact because RRM is not adiabatic across contacts, and a slight peak at the shock due to finite shock thickness.

Figure 23 shows test 1, which is Sod’s problem [3] with the initial conditions (*ρ*_l_, *u*_l_, *p*_l_) = (1.0, 0.0, 1.0) and (*ρ*_r_, *u*_r_, *p*_r_) = (0.125, 0.0, 0.1). The maximum error metrics for *ρ*, *u*, and *p* are (Δ_max *ρ*_, Δ_max *u*_, Δ_max *p*_) = (1.0e-5, 1.0e-3, 1.0e-3). The results are for time *t* = 1.5.

These results are typical of RRM, and match the Riemann solver’s results closely with two exceptions: the s-shaped contact, and the slight peak where the shock shows a finite thickness.

The s-shaped contact occurs because unlike a Riemann solver, RRM is not adiabatic across contacts, and models heat diffusion as a side effect of the algorithm. Wavefronts are created at contacts the same as at any other cell edges, so new cells are created across contacts, and gradual diffusion is the result.

We could easily make RRM adiabatic across contacts by adding a rule that when a tracer particle reaches a contact, its error metric is set to the maximum. This would insure that new cells are always created on one side of the contact or the other, keeping the contact sharp. We have not tried this yet, so RRM’s current behavior is more like a real fluid than a Riemann solver in this respect.

Shocks in RRM have a finite thickness that manifests as a thin peak at the shock front. The shock thickness decreases as the accuracy is increased. This is because RRM creates new cells at the shock front at a rate proportional to the accuracy, and the more frequently cells are created there, the more quickly the change in density, velocity, and pressure is propagated to the area behind the shock. In the limit of infinite accuracy, the shock would be infinitely thin as it is in the Riemann solver’s results.

Shocks in a real fluid also have a finite thickness of a few mean free paths, for a similar reason. It takes fluid atoms or molecules a few collisions to transition from their state in front of the shock to their state behind the shock. But because real fluids are not continuous, the shock thickness at a given set of conditions is essentially fixed by the fluid’s physical properties.

Unlike contacts, shocks in RRM will always be sharply defined, because they are formed by the edge of a supersonic cell pushing into slower fluid. Our wavefronts always travel at the local speed of sound *a*, so shocks are naturally self-forming because nearby wavefronts cannot outrun them.

### Test 2

**Figure 24:**
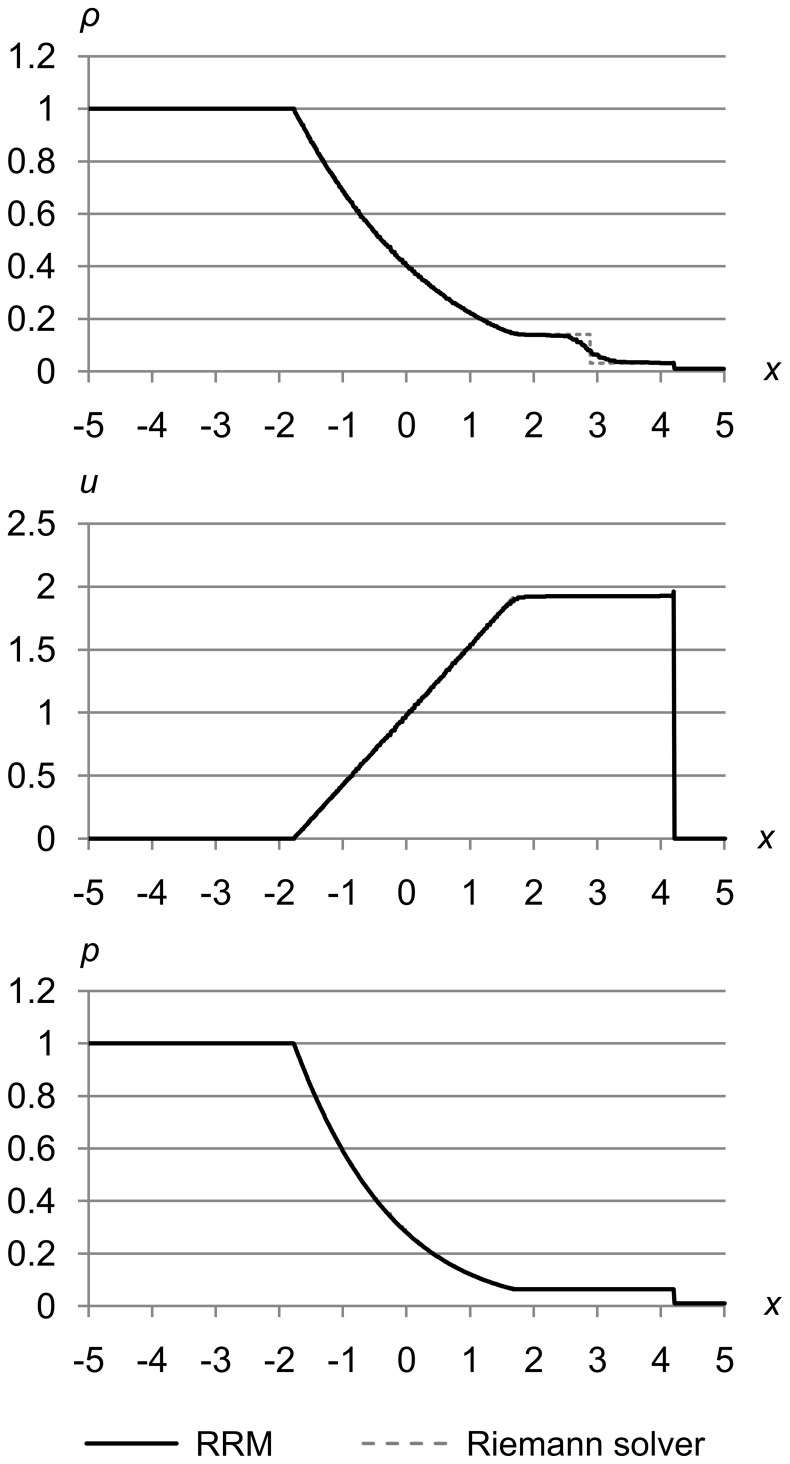
Test 2: Modified Sod’s test problem with 100-to-1 pressure differential. A modified version of Sod’s problem with initial conditions are (*ρ*_l_, *u*_l_, *p*_l_) = (1.0, 0.0, 1.0) and (*ρ*_r_, *u*_r_, *p*_r_) = (0.01, 0.0, 0.01), with maximum error metric (Δ_max *ρ*_, Δ_max *u*_, Δ_max *p*_) = (1.0e-4, 1.0e-4, 1.0e-3), at time *t* = 1.5. This test shows that RRM still gives good results on a problem that has a 100-to-1 pressure differential instead of the 10-to-1 differential of Sod’s problem.

Figure 24 shows test 2, which is a modified version of Sod’s problem due to Laney [19]. This problem has a 100-to-1 pressure differential instead of the 10-to-1 differential of Sod’s problem. The initial conditions are (*ρ*_l_, *u*_l_, *p*_l_) = (1.0, 0.0, 1.0) and (*ρ*_r_, *u*_r_, *p*_r_) = (0.01, 0.0, 0.01). The maximum error metric is (Δ_max *ρ*_, Δ_max *u*_, Δ_max *p*_) = (1.0e-4, 1.0e-4, 1.0e-3), and the results are for time *t* = 1.5.

This test shows that RRM can handle strongly supersonic flows. We can see that the contact is s-shaped as usual, and there is just a hint of a *u* peak at the shock front, but otherwise the results are in agreement with the Riemann solver. The velocity at the shock front is higher than in the original Sod’s problem, as we expect due to the greater pressure differential.

### Test 3

**Figure 25:**
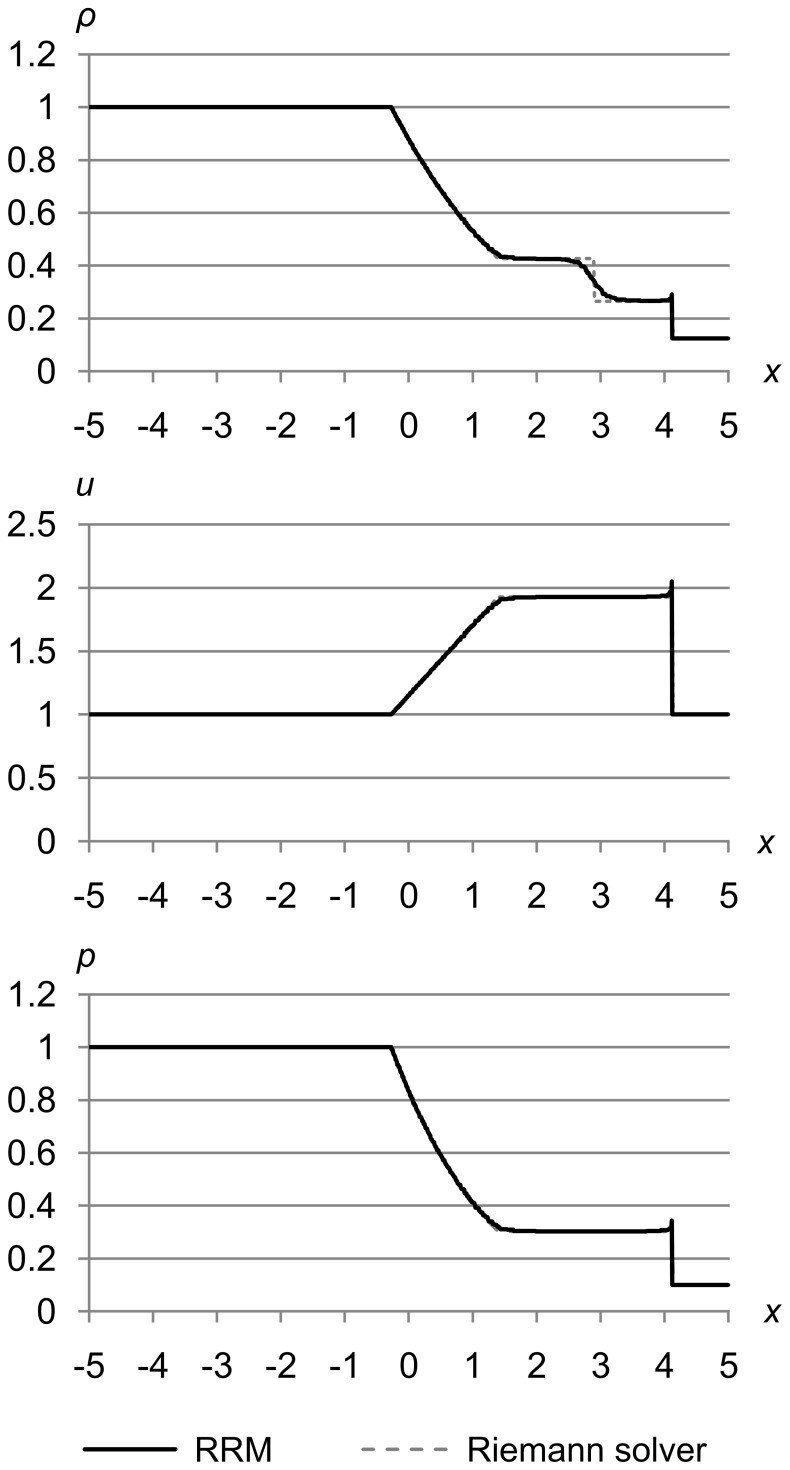
Test 3: Modified Sod’s test problem with initial *u* = 1.0. A modified version of Sod’s problem where the entire fluid moves right with *u* = 1.0, with initial conditions (*ρ*_l_, *u*_l_, *p*_l_) = (1.0, 1.0, 1.0) and (*ρ*_r_, *u*_r_, *p*_r_) = (0.125, 1.0, 0.1), with maximum error metric (Δ_max *ρ*_, Δ_max *u*_, Δ_max *p*_) = (5.0e-5, 1.0e-3, 1.0e-3), at time *t* = 1.5. This test shows one of the benefits of the fully Lagrangian nature of RRM. Since the cells all move to the right with *u* = 1.0, the shock front does not have to cross cell edges during the simulation, so the shock is just as sharp as in the *u* = 0 case.

Figure 25 shows test 3, which is a modified version of Sod’s problem where the entire fluid moves right with *u* = 1.0. The initial conditions are (*ρ*_l_, *u*_l_, *p*_l_) = (1.0, 1.0, 1.0) and (*ρ*_r_, *u*_r_, *p*_r_) = (0.125, 1.0, 0.1). The maximum error metric is (Δ_max *ρ*_, Δ_max *u*_, Δ_max *p*_) = (5.0e-5, 1.0e-3, 1.0e-3), and the results are for time *t* = 1.5.

This test shows one of the benefits of the fully Lagrangian nature of RRM. Since the cells all move to the right with *u* = 1.0, the shock front does not have to cross cell edges during the simulation, so the shock is just as sharp as in the *u* = 0 case. The *u* curve is identical to the *u* = 0 case, but shifted upwards by 1.0.

### Test 4

**Figure 26:**
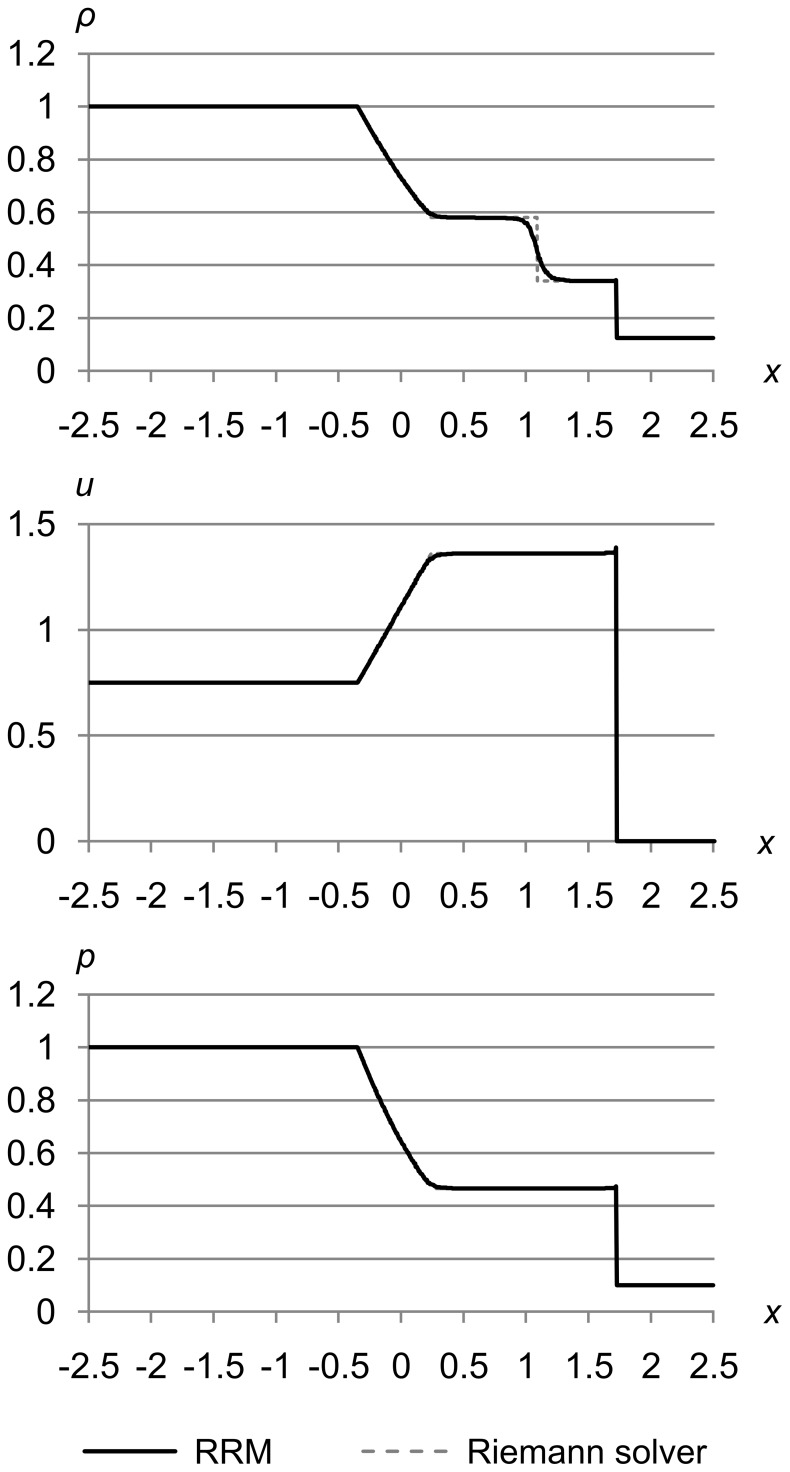
Test 4: Toro test 1. Toro’s test problem 1, with initial conditions (*ρ*_l_, *u*_l_, *p*_l_) = (1.0, 0.75, 1.0) and (*ρ*_r_, *u*_r_, *p*_r_) = (0.125, 0.0, 0.1), with maximum error metric (Δ_max *ρ*_, Δ_max *u*_, Δ_max *p*_) = (1.0e-5, 1.0e-4, 1.0e-4), at time *t* = 0.8. This test is similar to Sod’s problem, but the left cell is initially ramming into the right cell, so the velocity at the shock front is somewhat higher.

Figure 26 shows test 4, which is test problem 1 from page 225 of Toro’s book on Riemann solvers and numerical methods [1]. The initial conditions are (*ρ*_l_, *u*_l_, *p*_l_) = (1.0, 0.75, 1.0) and (*ρ*_r_, *u*_r_, *p*_r_) = (0.125, 0.0, 0.1). The maximum error metric is (Δ_max *ρ*_, Δ_max *u*_, Δ_max *p*_) = (1.0e-5, 1.0e-4, 1.0e-4), and the results are for time *t* = 0.8.

This test is similar to Sod’s problem, but the left cell is initially ramming into the right cell, so the velocity at the shock front is somewhat higher.

### Test 5

**Figure 27:**
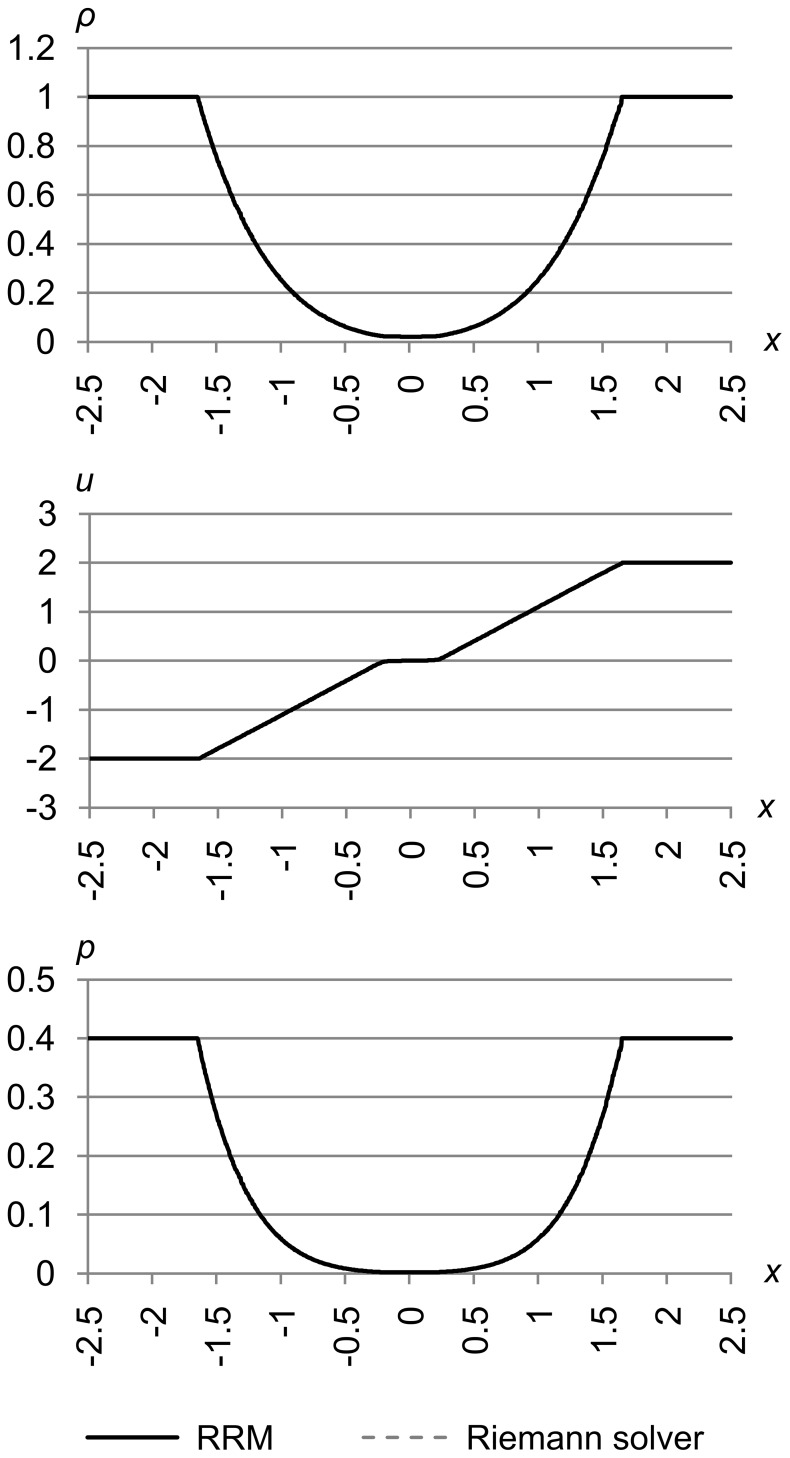
Test 5: Toro test 2. Toro’s test problem 2, with initial conditions are (*ρ*_l_, *u*_l_, *p*_l_) = (1.0, −2.0, 0.4) and (*ρ*_r_, *u*_r_, *p*_r_) = (1.0, 2.0, 0.4), with maximum error metric (Δ_max *ρ*_, Δ_max *u*_, Δ_max *p*_) = (1.0e-5, 1.0e-5, 1.0e-5), at time *t* = 0.6. This test shows that RRM can correctly handle the near-vacuum state created in the center.

Figure 27 shows test 5, which is test problem 2 from Toro’s book [1]. The initial conditions are (*ρ*_l_, *u*_l_, *p*_l_) = (1.0, −2.0, 0.4) and (*ρ*_r_, *u*_r_, *p*_r_) = (1.0, 2.0, 0.4). The maximum error metric is (Δ_max *ρ*_, Δ_max *u*_, Δ_max *p*_) = (1.0e-5, 1.0e-5, 1.0e-5), and the results are for time *t* = 0.6.

This test creates a near-vacuum in the center, which can cause problems in the iteration schemes that some Riemann solvers use to find *p*. RRM does not have any special difficulty with vacuum areas, either as part of the initial conditions, or evolved during the simulation as we see here.

Note that in this test we set Δ_max *u*_ relatively low. This is to resolve the velocity features near the origin that are far from the large density and pressure gradients on either side.

### Test 6

**Figure 28:**
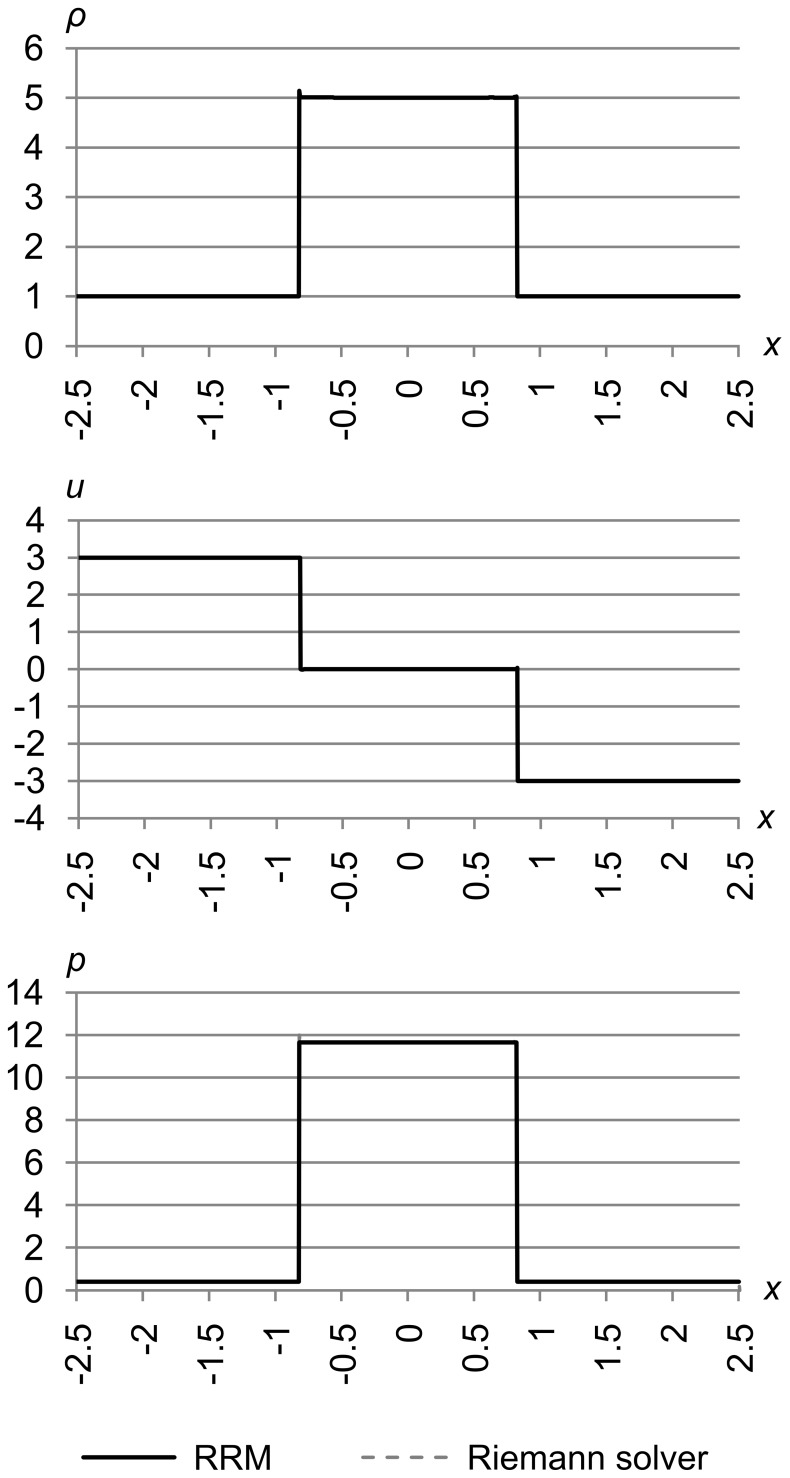
Test 6: Toro test 2 converging. A modified “converging” version of Toro’s test problem 2, with initial conditions (*ρ*_l_, *u*_l_, *p*_l_) = (1.0, 3.0, 0.4) and (*ρ*_r_, *u*_r_, *p*_r_) = (1.0, −3.0, 0.4), with maximum error metric (Δ_max *ρ*_, Δ_max *u*_, Δ_max *p*_) = (5.0e-4, 5.0e-4, 5.0e-4), at time *t* = 1.1. This is a test of symmetry and momentum conservation, to make sure that two colliding cells will pile up into one stationary mass with sharp edges.

Figure 28 shows test 6, which is a modified “converging” version of test problem 2 from Toro’s book [1]. The initial conditions are (*ρ*_l_, *u*_l_, *p*_l_) = (1.0, 3.0, 0.4) and (*ρ*_r_, *u*_r_, *p*_r_) = (1.0, −3.0, 0.4). The maximum error metric is (Δ_max *ρ*_, Δ_max *u*_, Δ_max *p*_) = (5.0e-4, 5.0e-4, 5.0e-4), and the results are for time *t* = 1.1.

This is a test of symmetry and momentum conservation, to make sure that two colliding cells will pile up into one stationary mass with sharp edges.

### Test 7

**Figure 29:**
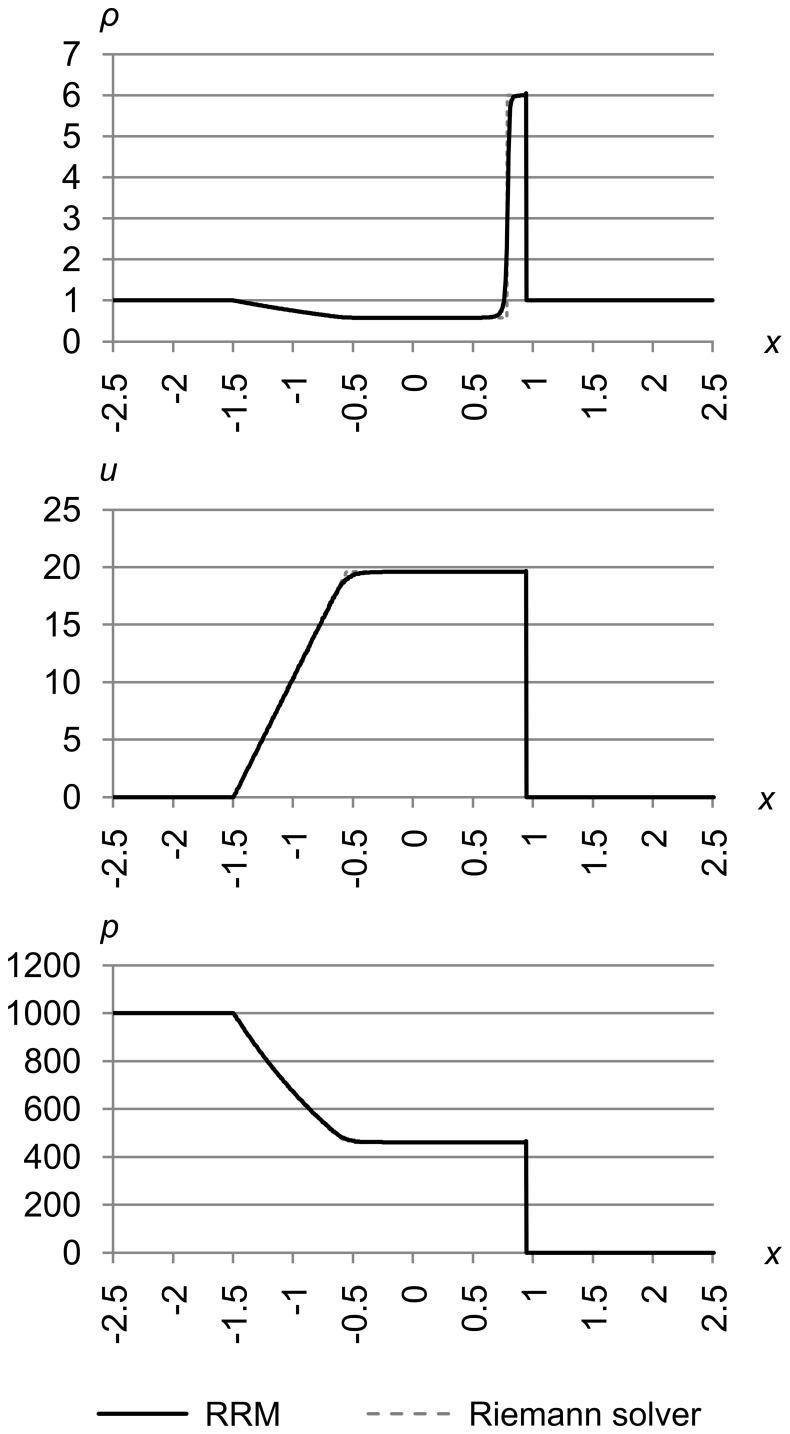
Test 7: Toro test 3. Toro’s test problem 3, with initial conditions (*ρ*_l_, *u*_l_, *p*_l_) = (1.0, 0.0, 1000.0) and (*ρ*_r_, *u*_r_, *p*_r_) = (1.0, 0.0, 0.01), with maximum error metric (Δ_max *ρ*_, Δ_max *u*_, Δ_max *p*_) = (1.0e-5, 5.0e-3, 1.0e-2), at time *t* = 0.04. This test’s solution contains a strong shock very close to a contact. Since RRM is spatially adaptive, it simply creates many new cells between the shock and the contact to get the required accuracy.

Figure 29 shows test 7, which is test problem 3 from Toro’s book [1]. The initial conditions are (*ρ*_l_, *u*_l_, *p*_l_) = (1.0, 0.0, 1000.0) and (*ρ*_r_, *u*_r_, *p*_r_) = (1.0, 0.0, 0.01). The maximum error metric is (Δ_max *ρ*_, Δ_max *u*_, Δ_max *p*_) = (1.0e-5, 5.0e-3, 1.0e-2), and the results are for time *t* = 0.04.

The solution to this test requires a strong shock to be placed very close to a contact. Since RRM is spatially adaptive, it simply creates many new cells between the shock and the contact to get the required accuracy.

### Test 8

**Figure 30:**
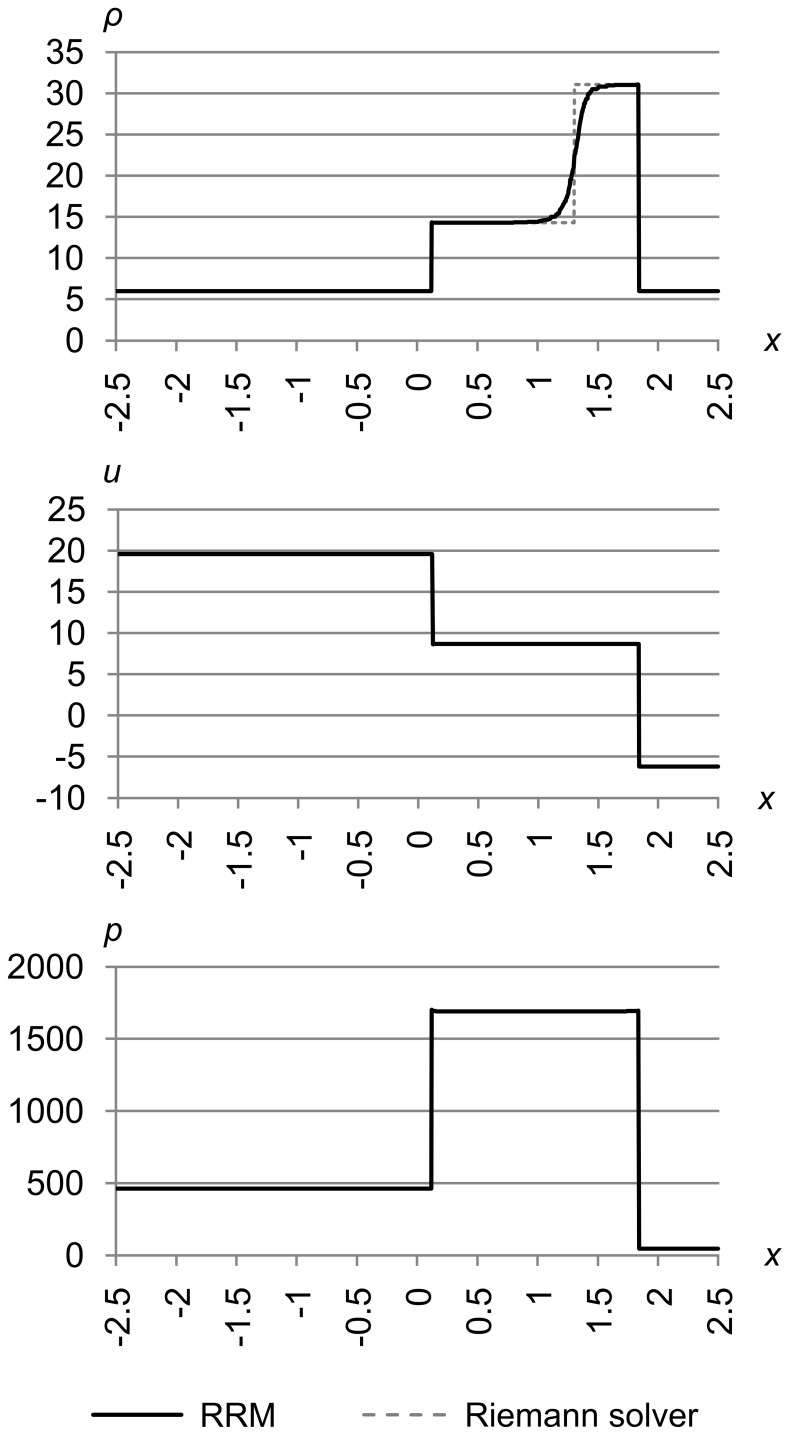
Test 8: Toro test 4. Toro’s test problem 4, with initial conditions (*ρ*_l_, *u*_l_, *p*_l_) = (5.99924, 19.5975, 460.894) and (*ρ*_r_, *u*_r_, *p*_r_) = (5.99242, −6.19633, 46.0950), with maximum error metric (Δ_max *ρ*_, Δ_max *u*_, Δ_max *p*_) = (5.0e-4, 1.0e-2, 1.0e-2), at time *t* = 0.15. The solution to this test has two rightward-traveling shocks with a contact between them. As usual, the shocks are sharply resolved and the contact is s-shaped due to RRM’s modeling of heat diffusion.

Figure 30 shows test 8, which is test problem 4 from Toro’s book [1]. The initial conditions are (*ρ*_l_, *u*_l_, *p*_l_) = (5.99924, 19.5975, 460.894) and (*ρ*_r_, *u*_r_, *p*_r_) = (5.99242, −6.19633, 46.0950). The maximum error metric is (Δ_max *ρ*_, Δ_max *u*_, Δ_max *p*_) = (5.0e-4, 1.0e-2, 1.0e-2), and the results are for time *t* = 0.15.

The solution to this test has two rightward-traveling shocks with a contact between them, which can be smeared out by some non-adaptive methods. As usual, the shocks are sharply resolved and the contact is s-shaped due to RRM’s modeling of heat diffusion.

### Test 9

**Figure 31:**
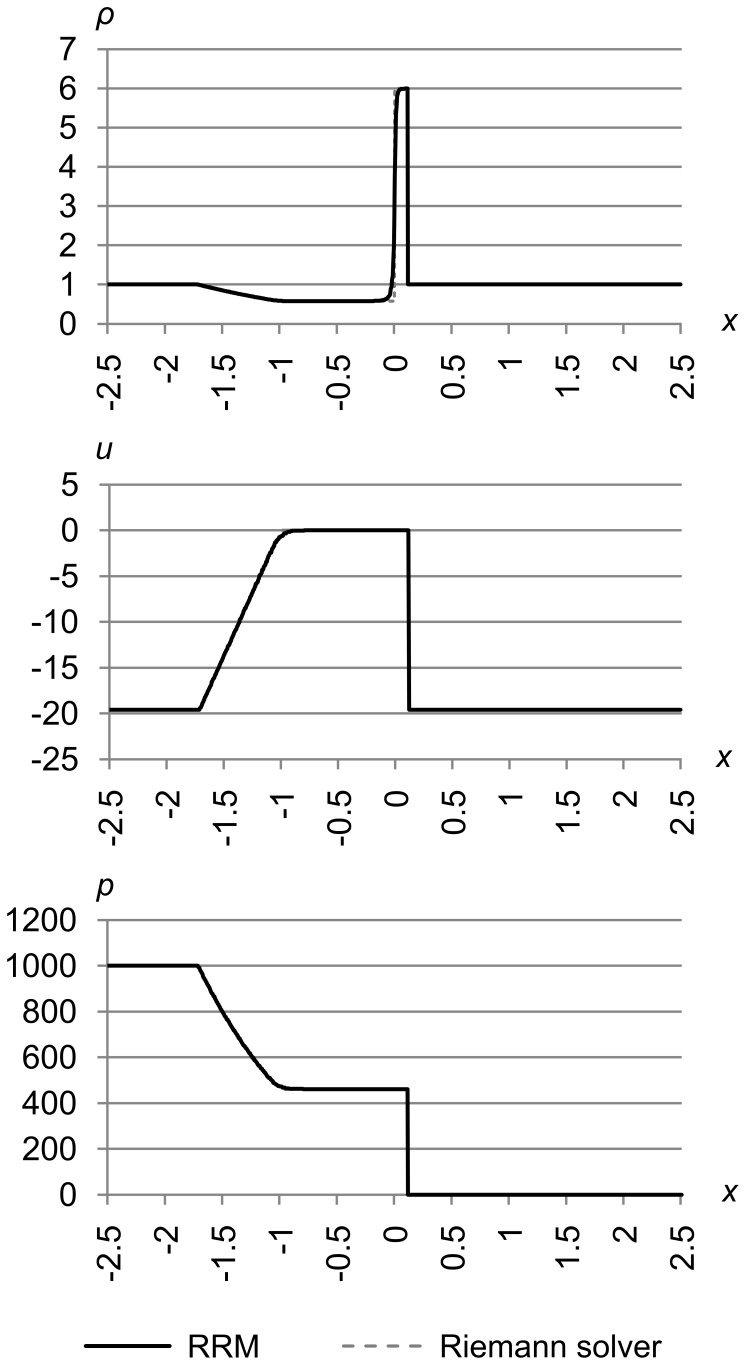
Test 9: Toro test 5. Toro’s test problem 5, with initial conditions (*ρ*_l_, *u*_l_, *p*_l_) = (1.0, −19.59745, 1000.0) and (*ρ*_r_, *u*_r_, *p*_r_) = (1.0, −19.59745, 0.01), with maximum error metric (Δ_max *ρ*_, Δ_max *u*_, Δ_max *p*_) = (1.0e-5, 1.0e-2, 1.0e-2), at time *t* = 0.03. The initial values of this test were designed to give an almost stationary contact at the origin, which causes difficulties for some numerical methods. RRM handles stationary contacts the same as it does moving contacts, due to the Lagrangian nature of the simulation.

Figure 31 shows test 9, which is test problem 5 from Toro’s book [1]. The initial conditions are (*ρ*_l_, *u*_l_, *p*_l_) = (1.0, −19.59745, 1000.0) and (*ρ*_r_, *u*_r_, *p*_r_) = (1.0, −19.59745, 0.01). The maximum error metric is (Δ_max *ρ*_, Δ_max *u*_, Δ_max *p*_) = (1.0e-5, 1.0e-2, 1.0e-2), and the results are for time *t* = 0.03.

The initial values of this test were designed to give an almost stationary contact at the origin, which causes difficulties for some numerical methods. RRM handles stationary contacts the same as it does moving contacts, due to the Lagrangian nature of the simulation.

### Absolute Error Analysis

To analyze RRM’s error as compared to a Riemann solver, first we will show qualitatively how the accuracy of the simulation decreases as the maximum error metrics are increased. Then we will define a quantitative measure of the error between RRM’s solution and that of the Riemann solver, and show how it decreases as each of the maximum error metrics is decreased. We will also show how the number of cells in the simulation increases as the error is reduced.

**Figure 32:**
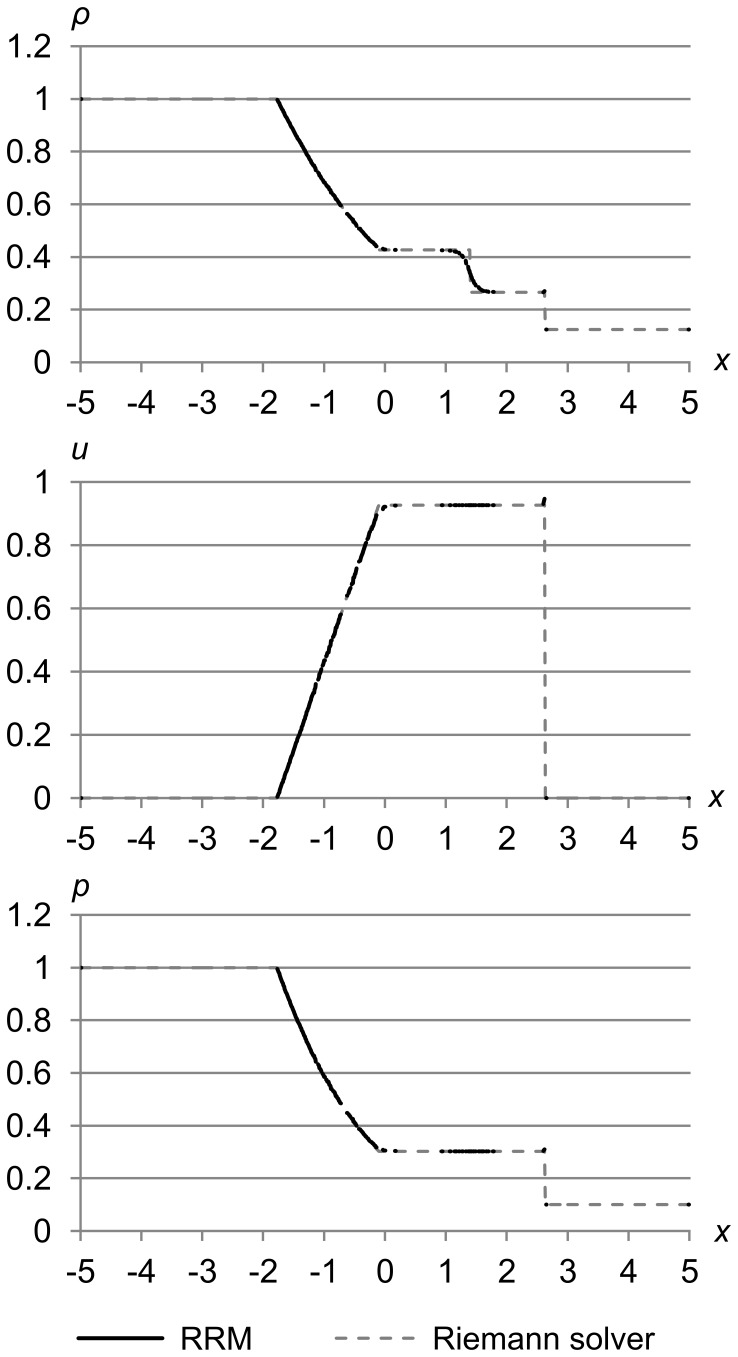
Sod’s test problem at high accuracy, showing cell density. Sod’s problem with initial conditions (*ρ*_l_, *u*_l_, *p*_l_) = (1.0, 0.0, 1.0) and (*ρ*_r_, *u*_r_, *p*_r_) = (0.125, 0.0, 0.1), with maximum error metric (Δ_max *ρ*_, Δ_max *u*_, Δ_max *p*_) = (1.0e-5, 1.0e-3, 1.0e-3), at time *t* = 1.5. Each of the approximately 800 cells is shown by a single dot, except the two edge cells which have two dots apiece. This figure shows that RRM is good at concentrating cells (and thereby computational effort) in areas of primitive variable gradient.

Figure 32 shows test 1 again, at the same accuracy as before, but this time only drawing one dot per cell (except for the edge cells, which have a dot on each side). The results are for time *t* = 1.5.

We can see that almost all of the approximately 800 cells are concentrated along the expansion fan and at the contact, with only one or two cells for each flat area. This illustrates how well RRM concentrates its computational effort on the active areas of the fluid.

**Figure 33:**
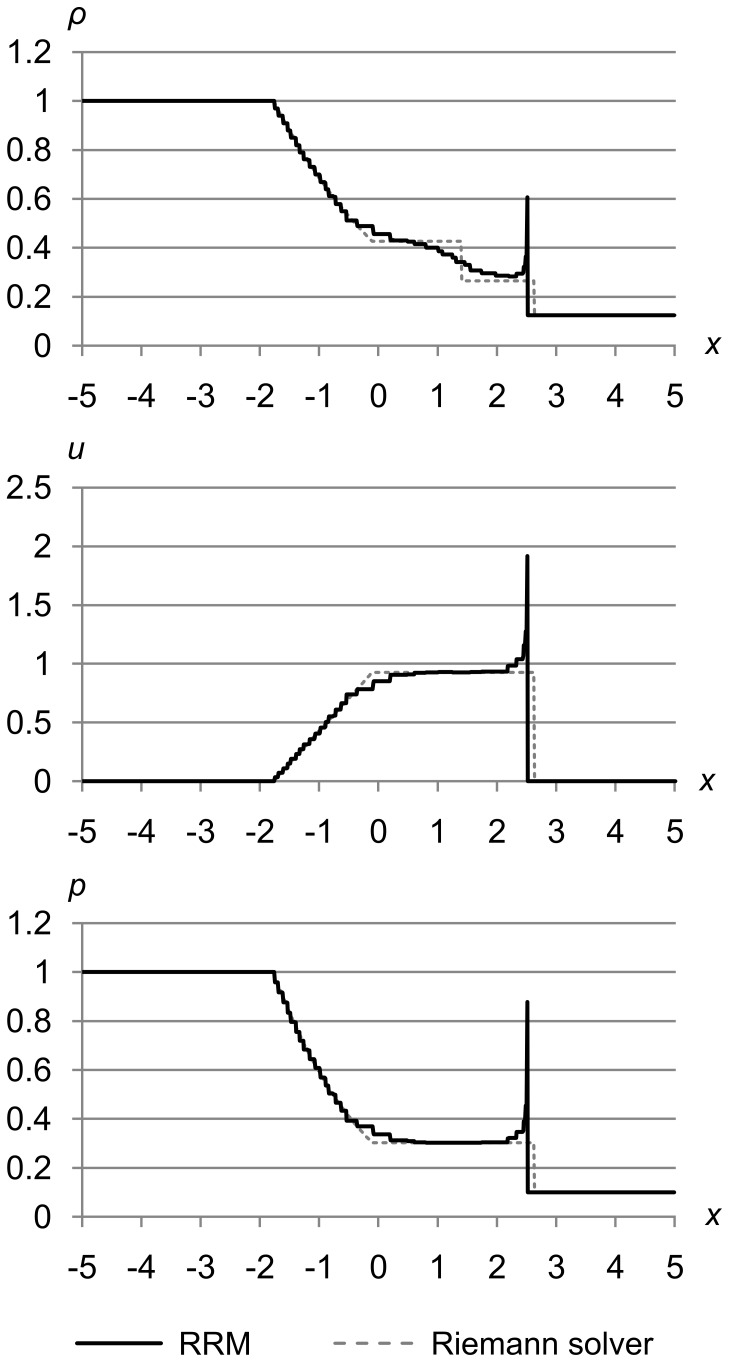
Sod’s test problem at medium accuracy. Sod’s problem with initial conditions (*ρ*_l_, *u*_l_, *p*_l_) = (1.0, 0.0, 1.0) and (*ρ*_r_, *u*_r_, *p*_r_) = (0.125, 0.0, 0.1), with the accuracy reduced by increasing the maximum error metric to (Δ_max *ρ*_, Δ_max *u*_, Δ_max *p*_) = (1.0e-3, 1.0e-2, 1.0e-2) to show how the simulation begins to degrade, at time *t* = 1.5. The shock is of increased thickness due to the lower accuracy, and is “blown back” from the correct location.

Figure 33 shows test 1 again, but with the accuracy reduced by increasing the maximum error metric to (Δ_max *ρ*_, Δ_max *u*_, Δ_max *p*_) = (1.0e-3, 1.0e-2, 1.0e-2) to show how the simulation begins to degrade. The results are for time *t* = 1.5.

At this accuracy, the widths of the fluid cells are directly visible in the jagged curve of the expansion fan, and the contact is mostly smeared out. As expected, the shock thickness is greater due to the decreased accuracy. The shock front is also “blown back” so that it trails a constant distance behind the correct location. This is because the shock in RRM is a dynamic phenomenon with no special-case code. If the accuracy is not set high enough, fluid will pile up at the shock front where it cannot be redistributed fast enough to maintain the correct wave shape.

**Figure 34:**
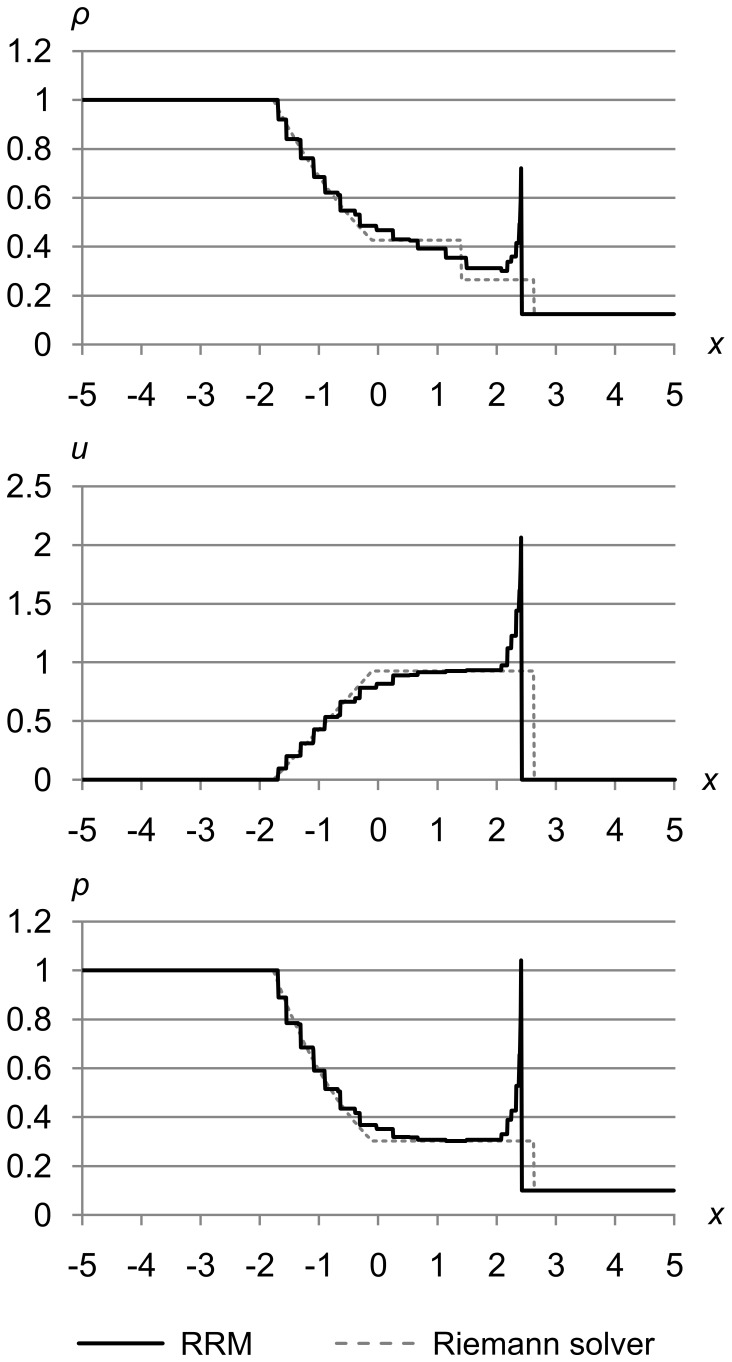
Sod’s test problem at low accuracy. Sod’s problem with initial conditions (*ρ*_l_, *u*_l_, *p*_l_) = (1.0, 0.0, 1.0) and (*ρ*_r_, *u*_r_, *p*_r_) = (0.125, 0.0, 0.1), with the accuracy further reduced by increasing the maximum error metric to (Δ_max *ρ*_, Δ_max *u*_, Δ_max *p*_) = (1.0e-2, 1.0e-2, 1.0e-2) to show a more extreme failure, at time *t* = 1.5. The shock is even thicker due to the lower accuracy, and is “blown back” even farther from the correct location.

Figure 34 shows test 1 yet again, but with the accuracy further reduced by increasing the maximum error metric to (Δ_max *ρ*_, Δ_max *u*_, Δ_max *p*_) = (1.0e-2, 1.0e-2, 1.0e-2) to show a more extreme failure. The results are for time *t* = 1.5.

The jagged expansion fan is even more pronounced here, since the cells are even larger. The contact is completely gone, the shock is blown back even further, and the spike at the shock front is even higher, since more fluid is piled up there.

Of course, we would never run a real simulation at such low accuracy. These figures are merely to show how the weaknesses of RRM differ from those of other methods. In particular, though RRM is a conservative method, that alone is does not guarantee correct shock placement as it does in FDM and FVM. But shocks in RRM remain very sharp even at very low accuracy, and there are no Gibbs oscillations near the shocks. This is because our cells are a piecewise-linear representation of the primitive variable values of the fluid.

Now we present a more quantitative analysis. We define.
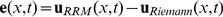
(19)to be the vector error between the primitive variable values in RRM’s solution and the primitive variable values in the Riemann solver’s solution. Then we define a maximum integral error norm
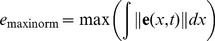
(20)to represent the maximum value, from the start time to some chosen end time, of the spatial integral of the norm of the error e(x,t) over the whole fluid.

Note that we choose the maximum integral error norm instead of the simpler maximum error norm.
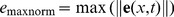
(21)because the maximum error norm for RRM is typically the thin peak right at the shock front, which is of almost constant height (though decreasing thickness) as simulation accuracy is increased.

**Figure 35:**
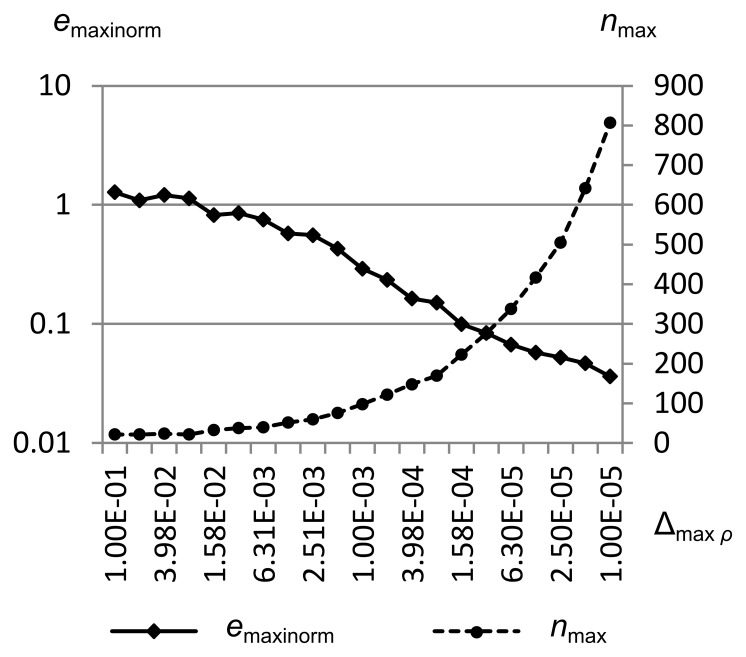
Integral error norm vs. maximum density error metric. Maximum integral error norm *e*_maxinorm_ and maximum number of cells *n*_max_ vs. the maximum density error metric Δ_max *ρ*_ for Sod’s problem. Δ_max *ρ*_ is swept from 1.0e-1 to 1.0e-5, while Δ_max *u*_ and Δ_max *p*_ are held constant at 1.0e-1. Simulation was from time *t* = 0.0 to *t* = 1.5. This figure shows that the error decreases logarithmically as the number of cells (and thus the computational effort) increases logarithmically.

Figure 35 shows *e*_maxinorm_ and the maximum number of cells *n*_max_ vs. the maximum density error metric Δ_max *ρ*_ for Sod’s problem. Δ_max *ρ*_ is swept from 1.0e-1 to 1.0e-5, while Δ_max *u*_ and Δ_max *p*_ are held constant at 1.0e-1. Simulation was from time *t* = 0.0 to *t* = 1.5.

We can see that once Δ_max *ρ*_ gets smaller than about 1.5e-4, the maximum number of cells in the simulation (which is a good proxy for the computational effort required) increases rapidly to maintain the approximately logarithmic decrease in maximum integral error norm. This computational effort goes into squaring off the contact, which is inherently diffusive in RRM.

Since *e*_maxinorm_ is integrated over a width of 10, *e*_maxinorm_ = 0.1 corresponds to an average absolute density error of 0.01, or about 1%. But the error is not evenly distributed. Most of the error is around the s-shaped contact, with a lesser amount near the shock front due to the transition region.

**Figure 36:**
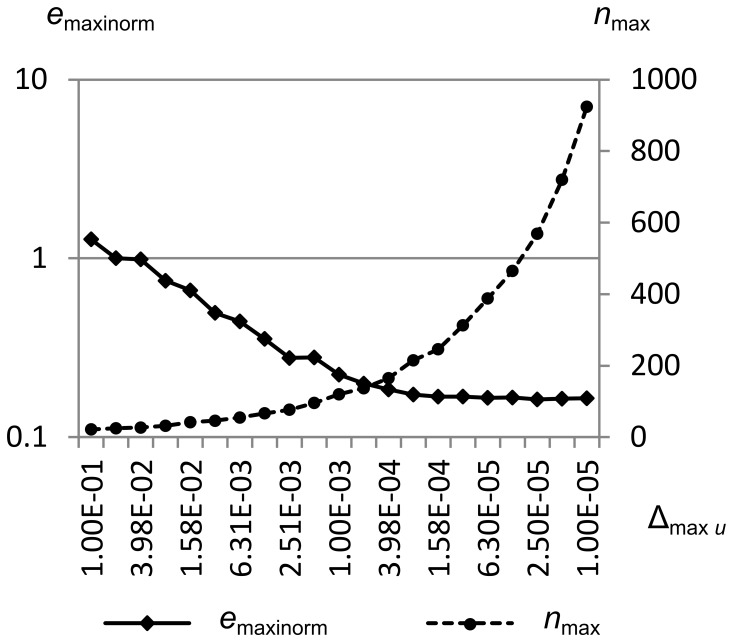
Integral error norm vs. maximum velocity error metric. Maximum integral error norm *e*_maxinorm_ and the maximum number of cells *n*_max_ vs. the maximum velocity error metric Δ_max *u*_ for Sod’s problem. Δ_max *u*_ is swept from 1.0e-1 to 1.0e-5, while Δ_max *ρ*_ and Δ_max *p*_ are held constant at 1.0e-1. Simulation was from time *t* = 0.0 to *t* = 1.5. This figure shows that the error cannot be decreased past a certain point solely by adjusting Δ_max *u*_, since there is little velocity gradient across the contact (where most of the error is concentrated in this test).

Figure 36 shows *e*_maxinorm_ and the maximum number of cells *n*_max_ vs. the maximum velocity error metric Δ_max *u*_ for Sod’s problem. Δ_max *u*_ is swept from 1.0e-1 to 1.0e-5, while Δ_max *ρ*_ and Δ_max *p*_ are held constant at 1.0e-1. Simulation was from time *t* = 0.0 to *t* = 1.5.

Note that we get less than a decade of decrease in maximum integral error norm as we decrease Δ_max *u*_, and for values lower than about 4.0e-4 we get very little additional benefit, though we increase computation effort by a factor of 5. This is because the velocity gradient across the contact is small, so decreasing Δ_max *u*_ will not cause more cells to be created there.

**Figure 37:**
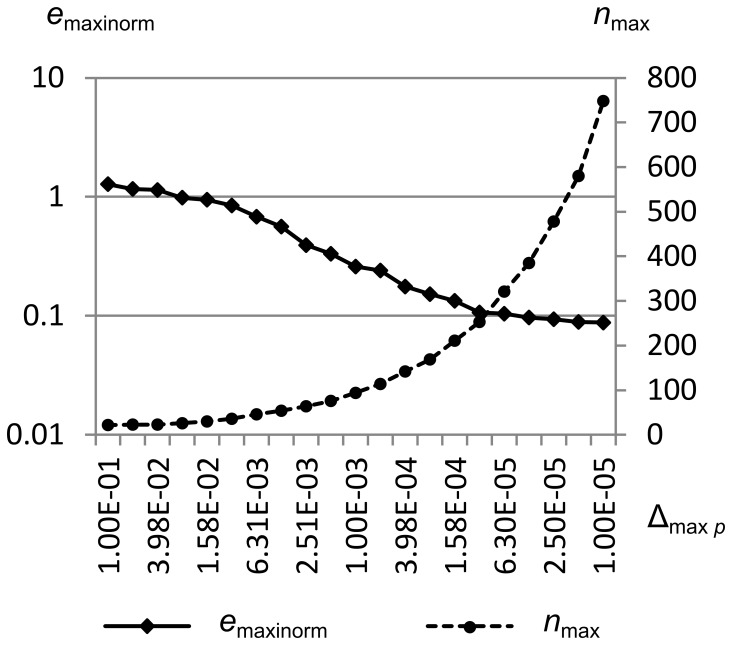
Integral error norm vs. maximum pressure error metric. Maximum integral error norm *e*_maxinorm_ and the maximum number of cells *n*_max_ vs. the maximum pressure error metric Δ_max *p*_ for Sod’s problem. Δ_max *p*_ is swept from 1.0e-1 to 1.0e-5, while Δ_max *ρ*_ and Δ_max *u*_ are held constant at 1.0e-1. Simulation was from time *t* = 0.0 to *t* = 1.5. This figure shows that the error cannot be decreased past a certain point solely by adjusting Δ_max *p*_, since there is little pressure gradient across the contact (where most of the error is concentrated in this test).

Figure 37 shows *e*_maxinorm_ and the maximum number of cells *n*_max_ vs. the maximum pressure error metric Δ_max *p*_ for Sod’s problem. Δ_max *p*_ is swept from 1.0e-1 to 1.0e-5, while Δ_max *ρ*_ and Δ_max *u*_ are held constant at 1.0e-1. Simulation was from time *t* = 0.0 to *t* = 1.5.

We see similar behavior to the Δ_max *u*_ sweep, where we get about one decade of decrease in the maximum integral error norm, with little further improvement as Δ_max *p*_ is reduced further. Again, this is because there is little pressure gradient across the contact, so reducing Δ_max *p*_ cannot improve the contact shape.

**Figure 38:**
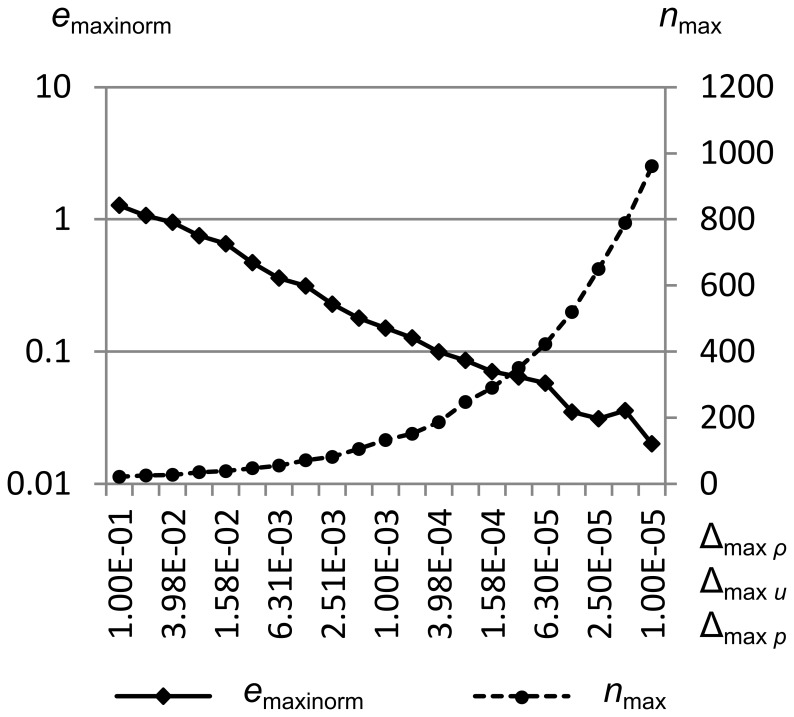
Integral error norm vs. all maximum error metrics. Maximum integral error norm *e*_maxinorm_ and the maximum number of cells *n*_max_ vs. all three maximum error metrics Δ_max *ρ*_, Δ_max *u*_, and Δ_max *p*_ for Sod’s problem. All three maximum error metrics are swept from 1.0e-1 to 1.0e-5 in tandem. Simulation was from time *t* = 0.0 to *t* = 1.5. This figure shows that there is a slight synergistic effect between the three maximum error metrics, since the minimum error achieved here is slightly lower than when each of the three is set to 1.0e-5 individually.

Figure 38 shows *e*_maxinorm_ and the maximum number of cells *n*_max_ vs. all three maximum error metrics Δ_max *ρ*_, Δ_max *u*_, and Δ_max *p*_ for Sod’s problem. All three maximum error metrics are swept from 1.0e-1 to 1.0e-5 in tandem. Simulation was from time *t* = 0.0 to *t* = 1.5.

This last graph shows that if all three maximum error metrics are decreased together, we can get a bit less error than if they are decreased separately. This indicates that there is some interaction between their effects, though it is small for this test case.

Taken together, the previous four figures demonstrate that for this particular test case, RRM’s error decreases almost logarithmically as Δ_max *ρ*_ is decreased logarithmically, whether by itself or in combination with the other maximum error metrics. However, other tests are sensitive to different maximum error metrics. For example, test 5 is sensitive to Δ_max *u*_, because its solution has velocity features near the origin that are far from the largest density or pressure gradients.

The maximum number of cells used by the simulation goes up almost logarithmically as the maximum error metrics are decreased logarithmically. And since the current RRM implementation performs O(*n*) operations per new cell created, where *n* is the number of cells in the fluid, this implies that the computational effort goes up logarithmically as well. This restricted our ability to run simulations with more than about 1000 cells in a reasonable time, which we define to be less than 5 minutes on one core of a 2.4 GHz Intel Core2 Quad CPU.

Future RRM implementations could perform as few as O(log *n*) or even O(1) operations per new cell created, if they used a more sophisticated data structure for cell intersection. We have so far avoided improving this data structure, since it makes the code much more difficult to maintain and alter for research purposes.

A note on the error-reducing efficiency of Δ_max *ρ*_, Δ_max *u*_, and Δ_max *p*_ in RRM is appropriate here. In the sweeps of Δ_max *u*_ and Δ_max *p*_ above, we see that the integral error norm decreases by fewer orders of magnitude than the number of cells increases by. This may simply show that decreasing the maximum error metrics of velocity and pressure does not efficiently reduce an error which is mostly in the density near the contact. Indeed, it appears that reducing Δ_max *ρ*_ reduces the integral error norm more efficiently, by approximately one order of magnitude as the number of cells increases from 100 to 1000. But for fewer than 100 cells, the error-reducing efficiency of Δ_max *ρ*_ is not as great. Overall, the error-reducing efficiency of the maximum error metrics in RRM is not yet fully understood.

These results indicate that RRM requires some further refinement if it is to efficiently produce results at any desired precision. If we assume the RRM implementation can be improved so that it performs only O(1) operations per cell, we would still like to insure that the number of cells always increases at a rate slower than the integral error norm decreases. One possibility is to adapt RRM to produce results more like those of a Riemann solver. As mentioned earlier, it would be straightforward to split new cells across contacts to maintain their sharpness, which would reduce a major source of error. Another possibility is to attempt to develop a new analytical solution to the Riemann problem that incorporates heat diffusion across contacts, and measure RRM’s error against that instead.

### Conservation Error Analysis

Conservation error is the difference between the conserved quantities currently present in all cells, and the original conserved quantities at the start of the simulation, assuming any boundary effects are properly accounted for. We define one conservation error for each conserved quantity:
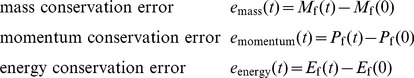
(22)where *M*_f_(*t*) is the total fluid mass, *P*_f_(*t*) is total fluid momentum, and *E*_f_(*t*) is total fluid energy, all functions of time.

**Figure 39:**
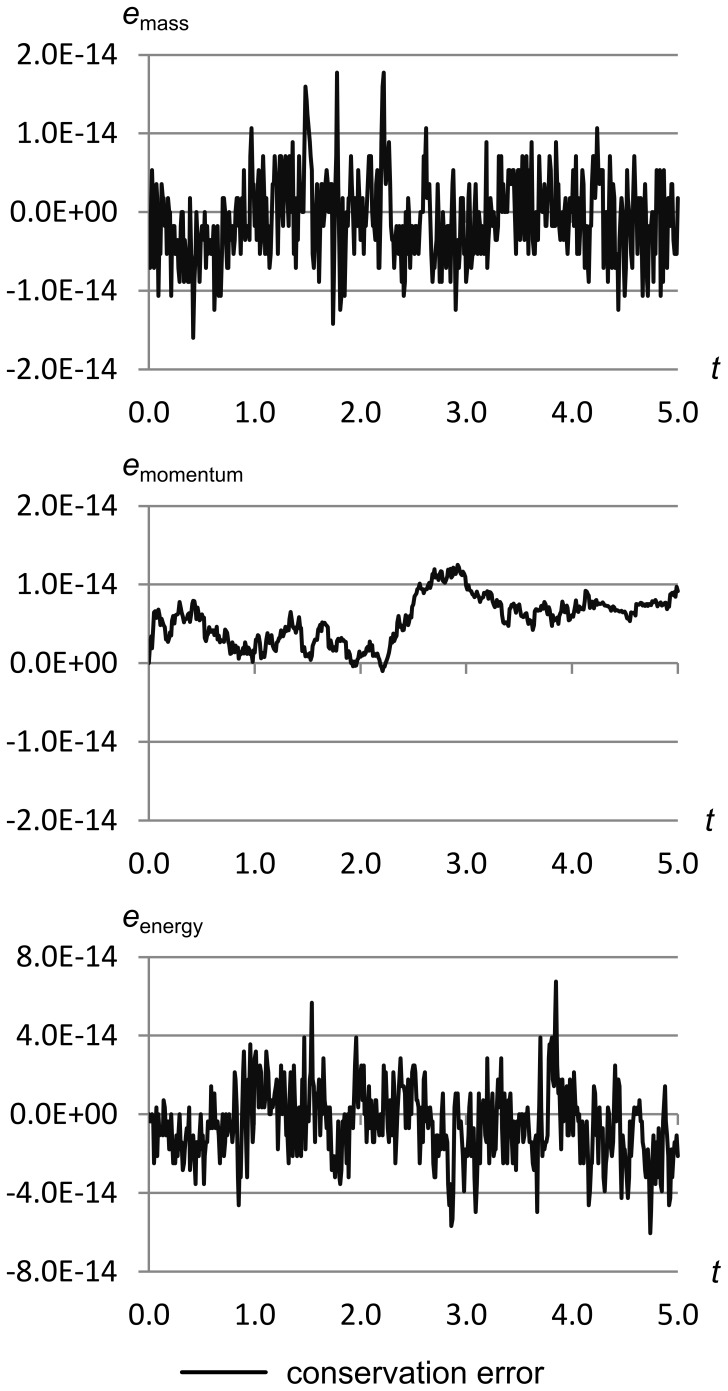
Conservation error vs. time on Sod’s problem over 5 seconds. The three conservation errors for Sod’s problem over the first five seconds of flow time, at a time resolution of 0.01 seconds. This graph shows that mass, momentum and energy are all conserved to within about ±6.0e-14. Total mass in the simulation is 11.25 kg, total momentum is 0.0 m·s, and total energy is 27.5 J.

Figure 39 shows the three conservation errors for Sod’s problem over the first five seconds of flow time, at a time resolution of 0.01 seconds.

This graph shows that mass, momentum and energy are all conserved to within about ±6.0e-14. Total mass in the simulation is 11.25 kg, total momentum is 0.0 m·s, and total energy is 27.5 J.

The first detail to note about this graph is that the conservation error is roughly two orders of magnitude larger than the floating-point precision ε_fp_, which on our test machine is about 2.22e-16 for 64-bit IEEE floating point.

This is due to our use of the primitive variable form of the Euler equations in the current RRM implementation. The conserved quantities are derived from the primitive variable values of cell width, density, velocity, and pressure in a series of floating-point operations, each of which may be incorrect by roughly ε_fp_. It takes only a few multiplicative operations for the error to grow to the observed value. Fortunately, since the signs of the individual errors are essentially random, the overall error does not tend to grow over time.

If we instead used the conservation form of the Euler equations in the RRM implementation, with a careful treatment we could get the error down to a smaller multiple of ε_fp_. But since the error is already small in an absolute sense and does not grow over time, we chose to stay with the primitive variable form because it is simpler to code.

The second detail to note about the conservation error graph is the overall trends of the lines. The mass and energy lines are as expected, with floating-point truncation error causing random fluctuation about the horizontal axis. However, the momentum line differs, showing instead a fluctuation around approximately *e*_momentum_ = 0.5e-14.

This is due to the initial conditions and time evolution of Sod’s problem. At time *t* = 0, the density and pressure are between 0.1 and 1.0, and calculating with these numbers to get mass and energy results in some nonzero error. However, the initial velocities are exactly zero, so initially *e*_momentum_ will also be exactly zero. As the simulation proceeds and cell velocities increase, the effective baseline of *e*_momentum_ is raised, since the calculations leading to momentum are no longer involve exact values of zero. Other test problems show variations on this behavior, but no problems tested so far show any time trend in conservation error.

### Other Boundary Conditions

So far we have considered only periodic boundary conditions, which are simple to implement since they do not affect the conserved quantities of the fluid. But RRM can handle many other types of boundary conditions by adjusting the conserved quantities of each new cell just before it is flattened. We will illustrate three more types of boundary conditions: solid, Dirichlet, and free.

**Figure 40:**
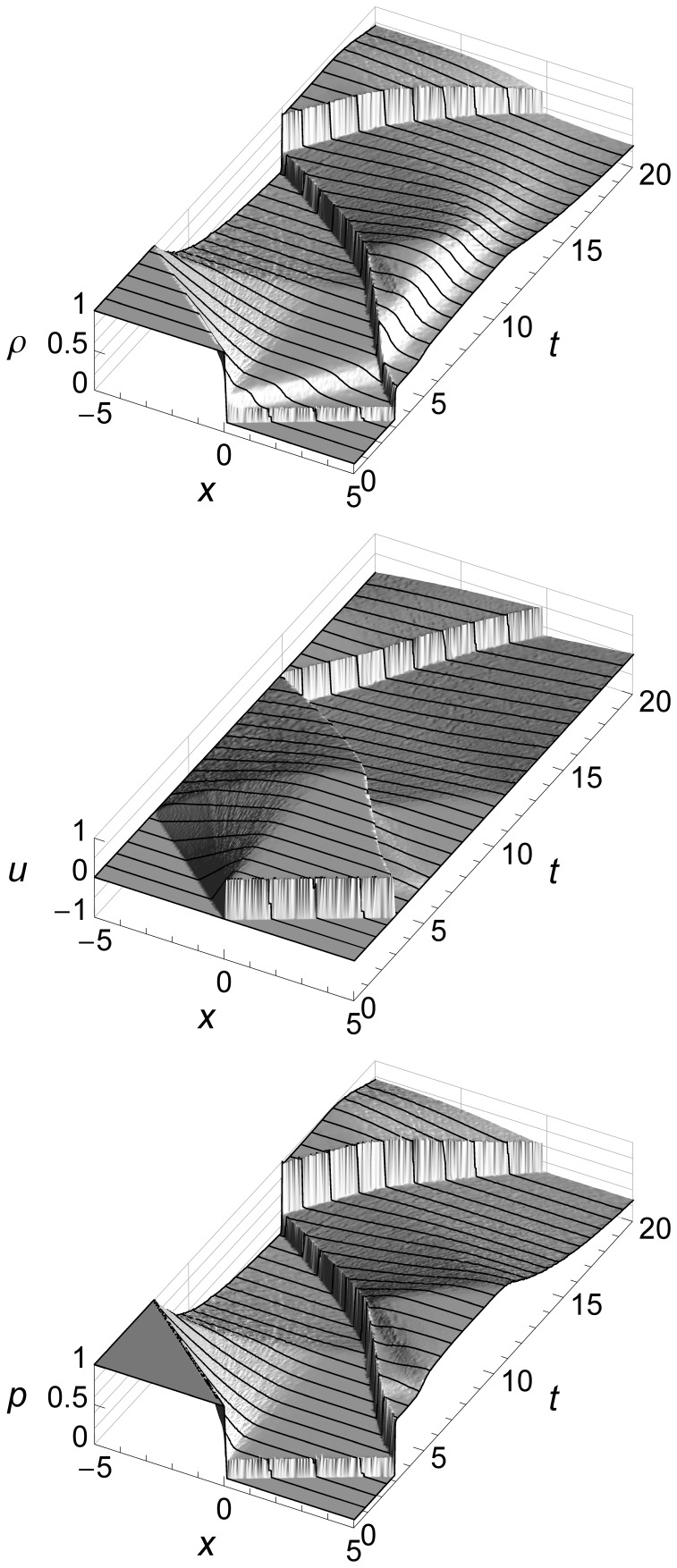
Sod’s problem with solid boundaries at *x* =  −5 and *x* = 5. Sod’s problem with solid boundaries at *x* = −5 and *x* = 5, from time *t* = 0 to *t* = 20. The initial conditions are (*ρ*_l_, *u*_l_, *p*_l_) = (1.0, 0.0, 1.0) and (*ρ*_r_, *u*_r_, *p*_r_) = (0.125, 0.0, 0.1). The maximum error metric is (Δ_max *ρ*_, Δ_max *u*_, Δ_max *p*_) = (1.0e-4, 1.0e-2, 1.0e-2). The shock wave hits the right boundary, reflects off it, and travels back across the fluid until it reflects off the left boundary.

Solid boundaries are immovable and impermeable. To make a solid boundary, we check if each new cell touches or crosses the boundary. If so, we set its momentum to zero, and adjust its width so its edge just touches the boundary. Figure 40 shows Sod’s problem with solid boundaries at *x* = −5 and *x* = 5, from time *t* = 0 to *t* = 20. The initial conditions are (*ρ*_l_, *u*_l_, *p*_l_) = (1.0, 0.0, 1.0) and (*ρ*_r_, *u*_r_, *p*_r_) = (0.125, 0.0, 0.1). The maximum error metric is (Δ_max *ρ*_, Δ_max *u*_, Δ_max *p*_) = (1.0e-4, 1.0e-2, 1.0e-2).

We can see the shock wave hit the right boundary, reflect off it, and travel back across the fluid until it reflects off the left boundary. If we let the simulation run indefinitely, the shock will travel back and forth many times, until numerical dissipation finally smooths it out. Eventually, the density and pressure will be flat, and the velocity will be everywhere zero.

**Figure 41:**
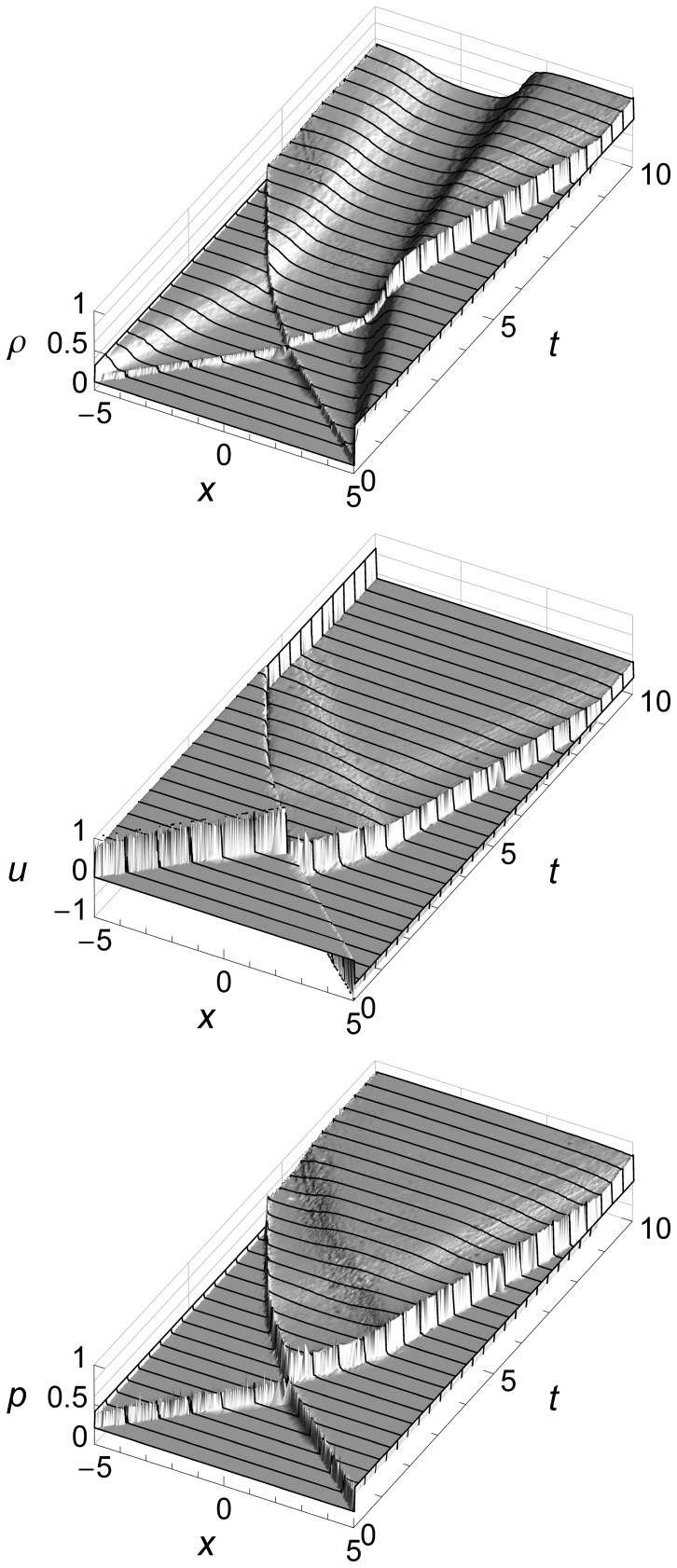
Inrush problem with Dirichlet boundaries at *x* =  −5 and *x* = 5. An inrush problem with Dirichlet boundaries at *x* = −5 and *x* = 5, from time *t* = 0 to *t* = 10. The initial conditions are (*ρ*_l_, *u*_l_, *p*_l_) = (0.1, 0.0, 0.2) and (*ρ*_r_, *u*_r_, *p*_r_) = (0.1, 0.0, 0.2). The boundary values are (*ρ*_l_, *u*_l_, *p*_l_) = (0.3, 0.6, 0.4) and (*ρ*_r_, *u*_r_, *p*_r_) = (0.6, −0.6, 0.5). The maximum error metric is (Δ_max *ρ*_, Δ_max *u*_, Δ_max *p*_) = (1.0e-4, 1.0e-3, 1.0e-3). Two asymmetrical shocks propagate in from the edges, cross near the center, and continue to the opposite edges, where they are squelched by the boundary conditions.

Dirichlet boundaries hold the primitive variable values of the fluid constant at the boundaries. To make a Dirichlet boundary, we check if each new cell touches or crosses a boundary. If so, we set its density, velocity, and pressure to some constant boundary values, and adjust its width so its edge just touches the boundary. Figure 41 shows an inrush problem with Dirichlet boundaries at *x* = −5 and *x* = 5, from time *t* = 0 to *t* = 10. The initial conditions are (*ρ*_l_, *u*_l_, *p*_l_) = (0.1, 0.0, 0.2) and (*ρ*_r_, *u*_r_, *p*_r_) = (0.1, 0.0, 0.2). The boundary values are (*ρ*_l_, *u*_l_, *p*_l_) = (0.3, 0.6, 0.4) and (*ρ*_r_, *u*_r_, *p*_r_) = (0.6, −0.6, 0.5). The maximum error metric is (Δ_max *ρ*_, Δ_max *u*_, Δ_max *p*_) = (1.0e-4, 1.0e-3, 1.0e-3).

We can see the two asymmetrical shocks propagate in from the edges, cross near the center, and continue to the opposite edges, where they are squelched by the boundary conditions. If we let the simulation run longer, the continuous fluid inflow fills the area higher and higher, with velocity everywhere zero, and density and pressure eventually becoming flat due to diffusion.

**Figure 42:**
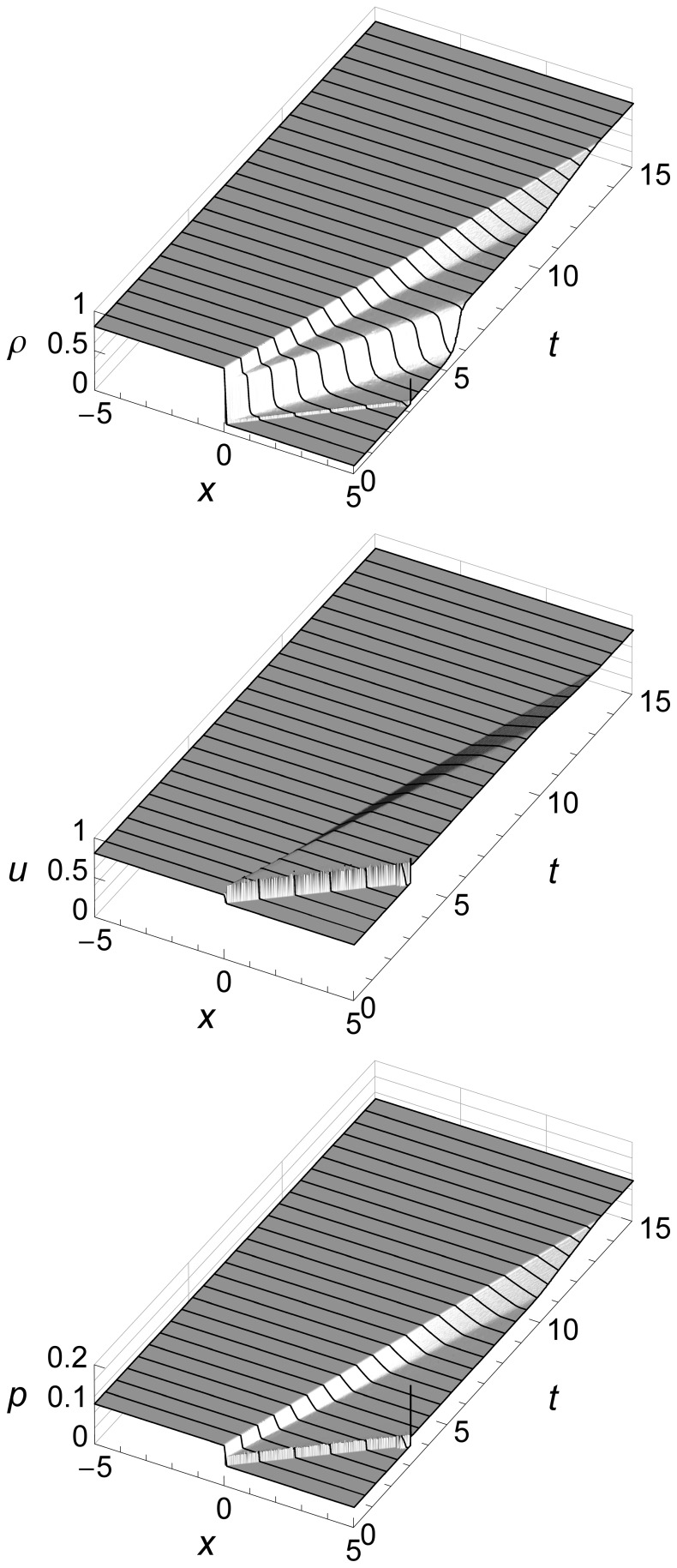
Rightward flow problem with far-field boundaries at *x* =  −5 and *x* = 5. A rightward flow problem with free boundaries at *x* = −5 and *x* = 5, from time *t* = 0 to *t* = 15. The initial conditions are (*ρ*_l_, *u*_l_, *p*_l_) = (0.8, 0.8, 0.1) and (*ρ*_r_, *u*_r_, *p*_r_) = (0.1, 0.7, 0.05). The maximum error metric is (Δ_max *ρ*_, Δ_max *u*_, Δ_max *p*_) = (5.0e-5, 1.0e-4, 1.0e-4). Fast-moving fluid flows in from the left boundary and pushes the slower fluid in front of it, forcing it out of the right boundary. After about *t* = 14 all of the fluid is in the left state, since all the right fluid as been pushed out.

Free boundaries let fluid flow in or out of the boundaries, without creating any disturbance that might propagate back into “interesting” parts of the fluid. To make a free boundary, we check if each new cell touches or crosses a boundary. If so, we set its density, velocity, and pressure to those of the intersected cell nearest the boundary (for inflow) or farthest from the boundary (for outflow), and adjust its width so its edge just touches the boundary. Figure 42 shows a rightward flow problem with free boundaries at *x* = −5 and *x* = 5, from time *t* = 0 to *t* = 15. The initial conditions are (*ρ*_l_, *u*_l_, *p*_l_) = (0.8, 0.8, 0.1) and (*ρ*_r_, *u*_r_, *p*_r_) = (0.1, 0.7, 0.05). The maximum error metric is (Δ_max *ρ*_, Δ_max *u*_, Δ_max *p*_) = (5.0e-5, 1.0e-4, 1.0e-4).

We can see that fast-moving fluid flows in from the left boundary and pushes the slower fluid in front of it, forcing it out of the right boundary. After about *t* = 14 all of the fluid is in the left state, since all the right fluid as been pushed out. Note that when the shock hits the right boundary at about *t* = 4, we can see a glitch. This is because if a cell is near the edge of the fluid, and there is no other cell between it and the edge, we extend it to touch the edge. In this case, it just happens to catch a narrow cell in the transition region and widen it so it is visible.

Finally, we mention two details that apply to all three boundary condition types discussed above. First, when producing 3D graphs of RRM simulations, we remove the very thin cells that can occur in the transition regions at shock fronts. This is simply to make the graphs more legible, since otherwise these very thin cells hide the details of the fluid behind them. You can see one of these cells at *t* = 4 on the right side of the very last graph above.

Second, we note that these adjustments to the conserved quantities of new cells require us to change the stored initial conserved quantities of the entire fluid by a commensurate amount, so the simulation will not fail its ongoing per-event conservation checks. This models the mass, momentum, and energy that are being added and removed at the boundaries.

### Source Terms

Our analysis so far has only treated the Euler equations in the homogeneous case, where no mass, momentum, or energy are added to or removed from the fluid during the simulation, except for a special case at the boundaries.

In the more general case, we augment the Euler equations with source terms thus:
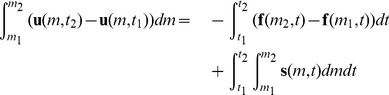
(23)using a new vector of source terms



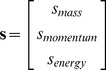
(24)Presenting a scheme to simulate these equations is beyond the scope of this paper. However, we can make a few remarks about how it might be possible.

A few cases would be simple. For example, mass or energy source terms that are constant in space and time could easily be implemented by adding mass or energy to new cells during flattening.

A few more cases are somewhat difficult, but feasible using splitting schemes similar to those described in chapter 15 of Toro’s book [1]. For example, momentum source terms that are constant in space and time could be implemented by changing the cells’ equations of motion from constant-velocity to varying-velocity, at the cost of complicating the intersection calculation of particles with cells.

The general case becomes very difficult. If spatially- and temporally-varying source terms are allowed, cells’ masses and energies could change in ways that would require both space and time integration to resolve at flattening time. The intersection calculation of particles with cells would also require solving ordinary differential equations, rather than simple algebraic equations.

## Discussion

So far we have shown that RRM gives correct results for many standard test problems, that RRM’s error decreases steadily as we increase the desired accuracy, and that RRM handles many common types of boundary conditions. Now we explain the similarities and differences between RRM and other CFD methods in detail, list some of RRM’s limitations, and suggest directions for future research.

### Comparison with Adaptive Eulerian Methods

Simple CFD methods advance time across the whole fluid in lockstep. But this wastes effort in smooth areas of the fluid, and gives suboptimal resolution in steep areas. To solve this problem, Osher and Sanders proposed locally varying time steps [20]. Such methods can make simulation much more efficient, but they require that special care be taken at the interfaces between areas of differing time resolution.

Adaptive mesh refinement (AMR) methods such as the one proposed by Berger and Oliger [21] flag points in the fluid with high estimated error for possible refinement. Then every so often, the flagged points are clustered together to determine the size, shape, and orientation of a new, finer sub-mesh to cover them. Finer sub-meshes are integrated using proportionately shorter time steps, so AMR is adaptive in both space and time. AMR requires special care when integrating the sub-meshes, to insure that the boundary conditions with the rest of the fluid maintain conservation.

RRM does not divide the fluid into areas of different mesh fineness or time step size. Instead, each new cell is created at its own individually chosen time, which need not bear any relation to the creation times of other cells. This gives us a very fine-grained spatio-temporal adaptivity, with the disadvantage that such a simulation must use an event queue instead of a simple time-stepping loop. RRM does not require a clustering algorithm, since new cells are preferentially created in high-gradient areas. But RRM does require the unioning of wavefronts before creating a new cell, which is a similar operation.

RRM differs from FDM and FVM in that it does not use numerical derivatives in the cell chopping and flattening process, only integrals. This means that RRM does not need a flux limiter or slope limiter to smooth spurious oscillations that can be caused by the extremely large gradients near shocks.

### Comparison with the Lattice Boltzmann Method

RRM differs from LBM firstly in that RRM is meshfree and Lagrangian, where LBM has a mesh (though it is called a lattice in LBM literature) and is Eulerian (since the fluid flows through fixed lattice sites). A more interesting difference between RRM and LBM is that in RRM, cells are free to move in any direction, where in LBM, fluid can only move in a fixed number of directions between adjacent sites. Fluid flows in LBM can therefore exhibit anisotropies, depending on the choice of lattice type and connectivity.

Cells in 2D and 3D RRM will have angular velocity, which will complicate the flattening process somewhat, where in LBM the collision process is much the same for all dimensionalities. Since collision takes place only at zero-size lattice sites, angular quantities do not arise, which keeps the programming simpler than RRM.

### Comparison with Adaptive Meshed Lagrangian Methods

To adapt to the time-varying features of a fluid, a meshed Lagrangian method can move the mesh relative to the fluid, change the mesh connectivity, or both.

The Arbitrary Lagrangian-Eulerian method (ALE) [22] combines the Eulerian and Lagrangian forms by creating a third “referential” coordinate system that is independent of both the fixed world coordinates and the moving material coordinates. This allows cells to move independently of both the fixed coordinate system and the material.

As an ALE simulation progresses in Lagrangian mode, the cells can be “rezoned” by allowing the mesh to move relative to the fluid, while keeping the same mesh connectivity. This rezoning helps keep the cells from becoming tangled or degenerate, which would prevent further simulation. Rezoning is an Eulerian process, since it allows fluid to flow across cell edges, and it can smear out contacts and shocks unless one is careful when rezoning near them. For this reason, if the fluid motion is complex enough, it may be impossible to keep the original mesh connectivity without unacceptably degrading accuracy.

The “free Lagrange” methods such as FLAG [23] and Whitehurst’s signal method [18] allow mesh points to be dynamically linked and unlinked over the course of the simulation, thereby changing the initial mesh connectivity as the fluid moves. These methods use a variety of heuristics to maintain a reasonable mesh, such as trying to keep nearest neighboring points connected, or trying to keep the angles of mesh triangles as equal as possible.

In RRM, there is no mesh connectivity, so there is no need to track or alter it over the course of simulation. RRM constantly creates new cells, which has an effect similar to rezoning in that it allows fluid to flow across the edges of chopped cells.

Moving finite element (MFE) methods [24]–[27] generalize the finite element method to better track moving fluid flow features using moving elements. MFE methods result in extremely stiff systems of ordinary differential equations (ODEs), and so require sophisticated implicit ODE solvers. They also require careful tuning with user-chosen parameters to keep the elements from becoming too small or bunching up at shocks. It is also possible to adaptively create and destroy nodes in an MFE method, as shown by Kuprat in 1992 [28].

Since each new RRM cell chops out and replaces what was underneath it, cells can never bunch up, and there is only one user-chosen parameter to set, the maximum error metric (though this metric does have three components). And since RRM does not use systems of equations, it does not need numerical solvers.

### Comparison with Previous Meshfree Methods

Of all current CFD methods, the meshfree methods are the most similar to RRM. Indeed, we categorize RRM itself as a meshfree method, since it shares the characteristics of being purely Lagrangian and not using a mesh. But as we will see, there are many differences between previous meshfree methods and RRM in how cells or particles are formed, how their motion is calculated, and how long they persist during a simulation.

Previous meshfree methods and RRM both discretize a fluid into a set of particles or cells with no connectivity between them. In previous meshfree methods like SPH and MPS, the particles are acted upon by forces over time, and thus change their velocities. In contrast, once a cell is created in RRM, it moves at a constant velocity even as parts of it are chopped away by the creation of subsequent new cells. This means RRM does not require integration of the equations of motion of its cells.

A major difference between RRM and SPH or MPS lies in how the primitive variable values like density and pressure are determined at each point in the fluid. SPH and MPS store the conserved quantities in moving material particles. These particles have zero extent, but a smoothing function allows us to find the primitive variable values in the spaces between the particles. In contrast, RRM does not store the conserved quantities of the fluid directly. Instead, they are the result of integrating the stored primitive variable values over the cell areas. Since the cells have non-zero sizes, and new cells are constantly being created to fill any gaps, we do not need a smoothing function. The tracer particles in RRM are non-material particles that do not carry any conserved quantities, they simply trace out the expanding acoustic waves in the fluid.

A particle-based meshfree method like SPH can be made adaptive by allowing the smoothing length to vary inversely with density, as shown by Benz in 1990 [29]. This is refined by Owen et al. in 1998 [30] to give each particle a time-varying, anisotropic smoothing length that attempts to keep the number of neighboring particles the same in each direction.

The motion of the tracer particles in RRM gives an effect similar to the use of anisotropic smoothing length in adaptive SPH, since the tracer particles sweep out an area that varies with the local speed of sound and the local fluid motion.

SPH can also be made adaptive by splitting and merging particles during simulation [31]–[33]. Splitting is done in low-density areas, and merging in high-density areas, to insure that the number of particles is appropriate to accurately track the fluid motion.

The previous meshfree method most similar to RRM is the Finite Mass Method (FMM) [34], [35]. FMM divides the fluid into finite-sized cells (called mass packets in FMM papers) with an internal distribution typically described by third-order B-splines. These cells can move, deform, and interact during simulation. If the cells become too deformed, the simulation is stopped, the fluid is remeshed into new, undeformed cells and the simulation is restarted.

Cells in RRM do not change shape, except in that parts of them are chopped away by the creation of new cells. Therefore RRM does not need to remesh to fix excessive cell deformation. RRM constantly creates and destroys cells, so any excessive bunching or gapping due to cell movement is fixed incrementally rather than all at once in a remeshing operation.

### Realm of Applicability

RRM should work for most systems of conservation equations that have a complete wave description, which are often described as hyperbolic. In one dimension, such a system typically has three types of waves: entropy waves, left-propagating acoustic waves, and right-propagating acoustic waves. In RRM, the motions of the cells model the entropy waves, and the moving tracer particles model the propagation of the acoustic waves.

RRM’s tracer particle methodology is useful only for compressible fluids, since incompressible fluids have no acoustic waves. So for example, RRM should work for the compressible Navier-Stokes equations, but not the incompressible Navier-Stokes equations.

RRM’s “chop out and flatten” methodology is most applicable to systems that are locally conservative. A system that is globally conservative but not locally conservative cannot easily be chopped out and flattened, since the flattening would require access to parts of the fluid other than those that were chopped out.

### Limitations

One limitation of RRM is that its data structures are complex and difficult to code correctly. Allowing cells to move and be chopped up over time is straightforward, but involves extra bookkeeping that many other methods do not require. Extension to 2D and 3D is possible, but will be even more complex since cells must be allowed to rotate as well as translate.

Another limitation of RRM is its use of event-driven simulation. This gives good spatial and temporal adaptivity, but it makes the algorithm more difficult to parallelize, especially on GPGPUs (General-Purpose Graphics Processing Units) such as NVIDIA’s Tesla where data-dependent branching is penalized.

A final limitation applies to von Neumann boundary conditions. Since RRM does not use spatial derivatives, it is difficult to hold them constant at the fluid boundaries. RRM could implement von Neumann boundary conditions with some difficulty by creating “ghost cells” just outside the boundaries to give the correct behavior, but it would not fit neatly into the “adjusting the conserved quantities of new cells” paradigm discussed in the above section on boundary conditions.

### Further Research

The obvious future research directions for RRM are extension to 2D and 3D, replacement of the Euler equations with the compressible Navier-Stokes equations, and parallelization. A not-so-obvious direction is using RRM to simulate the behavior of non-fluid fields containing inherent discontinuities or intractable nonlinearity. Since RRM does not evaluate numerical derivatives or solve systems of equations, it might be applicable to fields whose traditional discretizations are numerically ill-behaved or difficult to formulate.

RRM could also benefit from further investigation into its rate of convergence. As noted at the end of the section on absolute error, increases in computational effort do not always result in proportional decreases in the integral error norm. More research into this area would be helpful to insure that RRM can efficiently achieve any desired level of error.
